# Enhancing Human Skin Penetration with Biodegradable Enzymatic Nanomotors

**DOI:** 10.1021/acsnano.6c11619

**Published:** 2026-08-04

**Authors:** Carles Prado-Morales, Taco Waaijman, Inés Macías-Tarrío, Cristián Huck-Iriart, Tiziana Russo, Joël Gálvez-Savoca, Cesar Rodriguez-Emmenegger, Jasper J. Koning, Samuel Sánchez

**Affiliations:** † 284118 Institute for Bioengineering of Catalonia (IBEC), Barcelona Institute of Science and Technology (BIST), 08028 Barcelona, Spain; ‡ Universitat de Barcelona, Doctorate in Biotechnology, Facultat de Farmàcia i Ciències de l’Alimentació, 08028 Barcelona, Spain; § Department of Molecular Cell Biology and Immunology, 1209 Amsterdam UMC, Location VUMC, 1081 HV Amsterdam, The Netherlands; ∥ Departament de Bioquímica i Fisologia, Facultat de Farmàcia i Ciències de l’Alimentació, Universitat de Barcelona, 08028 Barcelona, Spain; ⊥ ALBA Synchrotron Light Source, Cerdanyola del Vallès, 08290 Barcelona, Spain; # Universitat de Barcelona, Facultat de Biologia, 08028 Barcelona, Spain; ¶ DWI − Leibniz Institute for Interactive Materials, 52074 Aachen, Germany; ∇ Institució Catalana de Recerca i Estudis Avaņats (ICREA), 08010 Barcelona, Spain; ○ Amsterdam institute for Immunology and Infectious diseases, Inflammatory diseases, 1081 HV Amsterdam, The Netherlands

**Keywords:** nanomotors, nanobots, nanomedicine, biological barrier, skin, transdermal delivery, human skin model

## Abstract

The skin is the body’s primary biological barrier, largely due to the highly organized structure of the stratum corneum (SC). Although essential for protection, this barrier function limits the efficacy of transdermal drug delivery, as most topically applied compounds fail to reach deeper skin layers at therapeutically relevant concentrations. Existing approaches often rely on physical disruption of the barrier, which can cause undesirable side effects. Moreover, many prior studies have been conducted in murine models, which do not accurately recapitulate human skin physiology, hindering the translation to humans. Here, we present an alternative approach using enzymatically powered nanomotors tested in a human reconstructed skin model. We developed organic nanomotors composed of poly­(lactic-*co*-glycolic acid) functionalized with urease, and we proved their biocompatibility and degradability. Our results show that nanomotors penetrate the skin with 15.7% efficacy, 2.5 times more than passive nanoparticle controls. This enhanced penetration is attributed to their active motion and their ability to induce alterations in the lipid organization of the SC, an effect confirmed by synchrotron radiation small-angle X-ray scattering and electron microscopy. These findings highlight the potential of enzymatic nanomotors as a nondisruptive and effective platform for future transdermal delivery in human skin, combining advantages of both chemical enhancers and nanoparticles.

## Graphical abstract

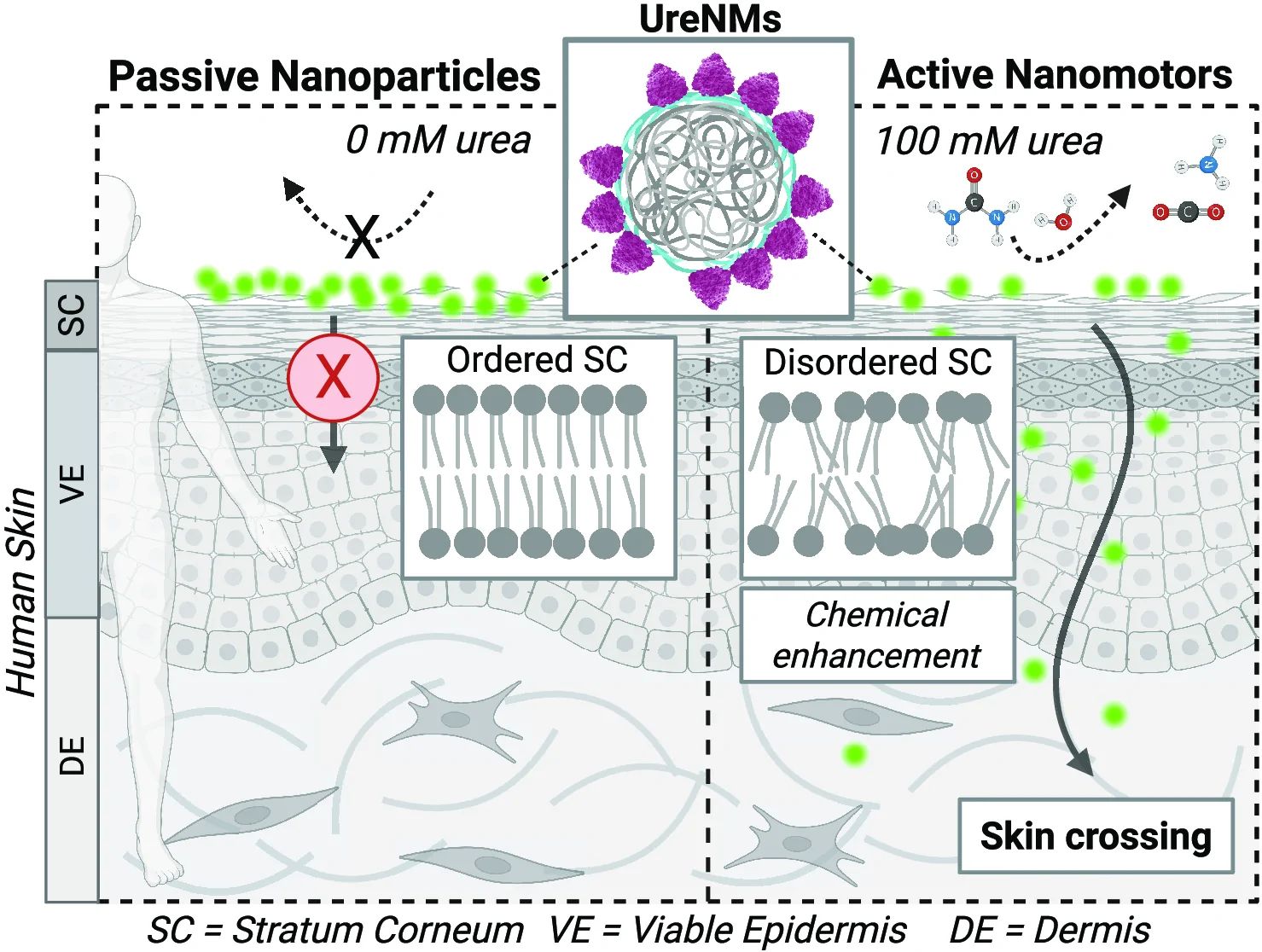

## Introduction

The skin, as the body’s outermost line of defense, is constantly exposed to environmental threats. Its complex architecture serves as one of the primary biological barriers for protecting the body.^1^ The stratum corneum (SC), the superficial layer of the epidermis, is primarily responsible for this barrier function.^2–5^ Skin conditions are among the most prevalent disorders worldwide, provoking substantial burden and notable costs on healthcare systems.^6,7^ The efficient therapy of topically administered active pharmaceutical ingredients (APIs) is significantly limited due to the SC architecture and skin protective role.^8^ Accordingly, extensive research has focused on the development of transdermal drug delivery technologies to enhance the effectiveness of topical treatments. To date, three generations of transdermal technologies have been developed, including the chemical modification of small APIs, the physical disruption of the epidermis, the use of chemical penetration enhancers, and the design of specialized nanoparticles (NPs).^2,4,9–11^ The use of chemical enhancers relies on altering the lipid organization of the SC.^12,13^ Nevertheless, this approach is a concentration-dependent method, ineffective at particular concentrations and that must be adjusted for each specific API.^3,9^ In contrast, NPs have attracted considerable attention due to their capacity to enhance the bioavailability of a broad spectrum of small drugs and biologic APIs.^14,15^ However, NPs encounter the same diffusional limitation as conventional drugs when targeting skin.^16^ Although NPs can follow the follicular route, the limited surface area of skin appendages (0.1%) renders this route negligible for most biomedical purposes.^4,5,17^ Consequently, effective therapeutic delivery often requires combining NPs with physical disruption techniques.^16,18^ Only a limited number of ultraflexible deformable vesicles have been reported to cross the epidermis by *trans*- and intercellular routes.^9,11^ Nevertheless, their inherent chemical composition results in poor stability, high cost, complex manufacturing, and a lack of batch-to-batch reproducibility and restricts their drug-loading capacity.^9,19^

To address these limitations, recent attention has shifted toward active delivery systems known as nanomotors (NMs). NMs are self-propelled NPs that can actively move by transforming various forms of energy, such as light, magnetic, or chemical, into mechanical force. These self-propelled NPs have emerged as the next-generation advanced carriers due to their enhanced ability to navigate through complex biological environments while simultaneously protecting and delivering APIs.^20,21^ Among the various propulsion mechanisms, enzyme-powered NMs are considered one of the most promising alternatives for overcoming biological barriers.^22–26^ These NMs consist of an NP asymmetrically functionalized with enzymes.^27^ By consuming available fuels, they can convert chemical energy into mechanical motion, effectively acting as nanoscale motors that propel the particles through their environment.^28–32^ To date, only light-powered NMs requiring external control have been evaluated as transdermal drug delivery enhancers.^33–35^ These systems have relied on inorganic or complex chassis and have been tested exclusively in murine skin models, which fail to capture the structural characteristics of human skin.^36^ Therefore, there remains a clear need to develop an organic degradable design validated by using a reliable human skin model.

Skin crossing experiments are performed in animal models to mimic the high complexity of human tissues. Nevertheless, porcine and murine models do not fully reproduce the characteristics of human skin.^37–39^ Porcine skin most accurately mimics the human architecture, but mechanical differences and ex vivo sample preparation procedures cause an abnormal increase in permeability.^37,38^ On the other hand, murine models, which are the most widely used, exhibit major anatomical differences, representing an important translational gap.^33–35,39,40^ Furthermore, increasing ethical concerns have prompted the development of human skin models as alternative in vitro testing platforms. Human skin equivalent technology has undergone significant advancements over the years.^41,42^ These models successfully replicate the architecture of the dermal and epidermal layers. Notably, the barrier function closely resembles that of native human skin due to the preservation of the SC’s lipid lamellar organization.^43^ The absence of hair follicles, which eliminates interference from appendage routes, together with a native-like SC, makes the in vitro human skin model an ideal candidate for testing the ability of NMs to overcome the human skin barrier.

In this study, we explored the use of PLGA-NMs combined with urease (UreNMs) as a delivery vehicle capable of crossing human skin, offering an alternative strategy that merges the advantages of NP carriers with those of local chemical enhancers. The use of PLGA as a chassis was selected for its simple synthesis, biocompatibility, biodegradability, and regulatory acceptance to be used in clinics.^44^ Furthermore, PLGA NPs are a well-established and tunable platform for delivering a wide range of APIs, including small molecules, proteins, and genetic material.^45,100^ Specifically, in the context of skin conditions, PLGA NPs have been successfully loaded with both antiskin cancer and anti-inflammatory APIs.^46–49^ Urease was chosen for two primary reasons: it provides enzymatic propulsion for active NM motion, and its substrate (urea) is a well-established compound in dermatological topical applications.^50,51^ To validate our system, we used a model that accurately reconstructs native human skin to observe the enhanced transdermal penetration of active UreNMs by the transepidermal route. After demonstrating the crossing capabilities of our technology, we investigated the mechanism in detail using an advanced technique: synchrotron radiation small-angle X-ray scattering (SAXS), revealing alterations in the lipid organization of the SC due to the locally increased pH produced by the UreNMs’ catalytic reaction. In addition, electron microscopy images of the SC revealed increased intercorneocyte spaces following incubation with UreNMs. Altogether, these findings provide new insights into the mechanisms by which active urease-NMs can overcome the human skin barrier, paving the way for advanced transdermal drug delivery systems.

## Results and Discussion

### PLGA UreNMs Synthesis and Characterization

PLGA NPs were synthesized by an oil-in-water emulsion method, in which the organic phase (ethyl acetate) contained the PLGA polymer, while the surfactant Mowiol was present in the aqueous phase. Scanning and transmission electron microscopy (SEM and TEM) images show the resulting spherical PLGA NPs with a mean core diameter of 126.76 ± 22.25 nm (Figures 1B,D and S1C,D,F). The mean hydrodynamic diameter increased to 177.48 ± 13.47 nm, due to the absence of vacuum during sample preparation and the hydrated state of the particles during measurement (Figure S1E). Dynamic light scattering (DLS) further reflects the lack of aggregation in the resulting NPs’ population with a polydispersity index (PDI) of 0.038 ± 0.03 (Figure 1I). The scheme illustrated in Figure 1A was employed to transform the PLGA NPs into NMs. Accordingly, the negatively charged PLGA­(−COOH) core was covered by electrostatic deposition of a positive layer of polyethylenimine (PEI), thereby introducing surface-exposed amine groups (NH_2_). The assembly of the PEI layer was confirmed by a shift in the surface charge, from −12.91 ± 1.45 mV (PLGA) to 45.68 ± 3.35 mV (PLGA–PEI) (Figure 1J). The PEI coating was optimized to maximize positive charge while minimizing the amount of polymer applied (Figure S1A,B). TEM images further revealed an enhanced border contrast of the PLGA–PEI NPs when compared to the PLGA particles (Figures 1E,F and S1F,G). The thin layer observed in the PLGA particles arises from artifacts associated with the contrast agent uranyl acetate (Figures 1E and S1F).^52^ In the case of PLGA–PEI NPs, the thicker border detected may be attributed to modified interactions between the PEI layer and the contrast agent, consistent with the successful deposition of the coating (Figures 1F and S1G).

**Figure 1:**
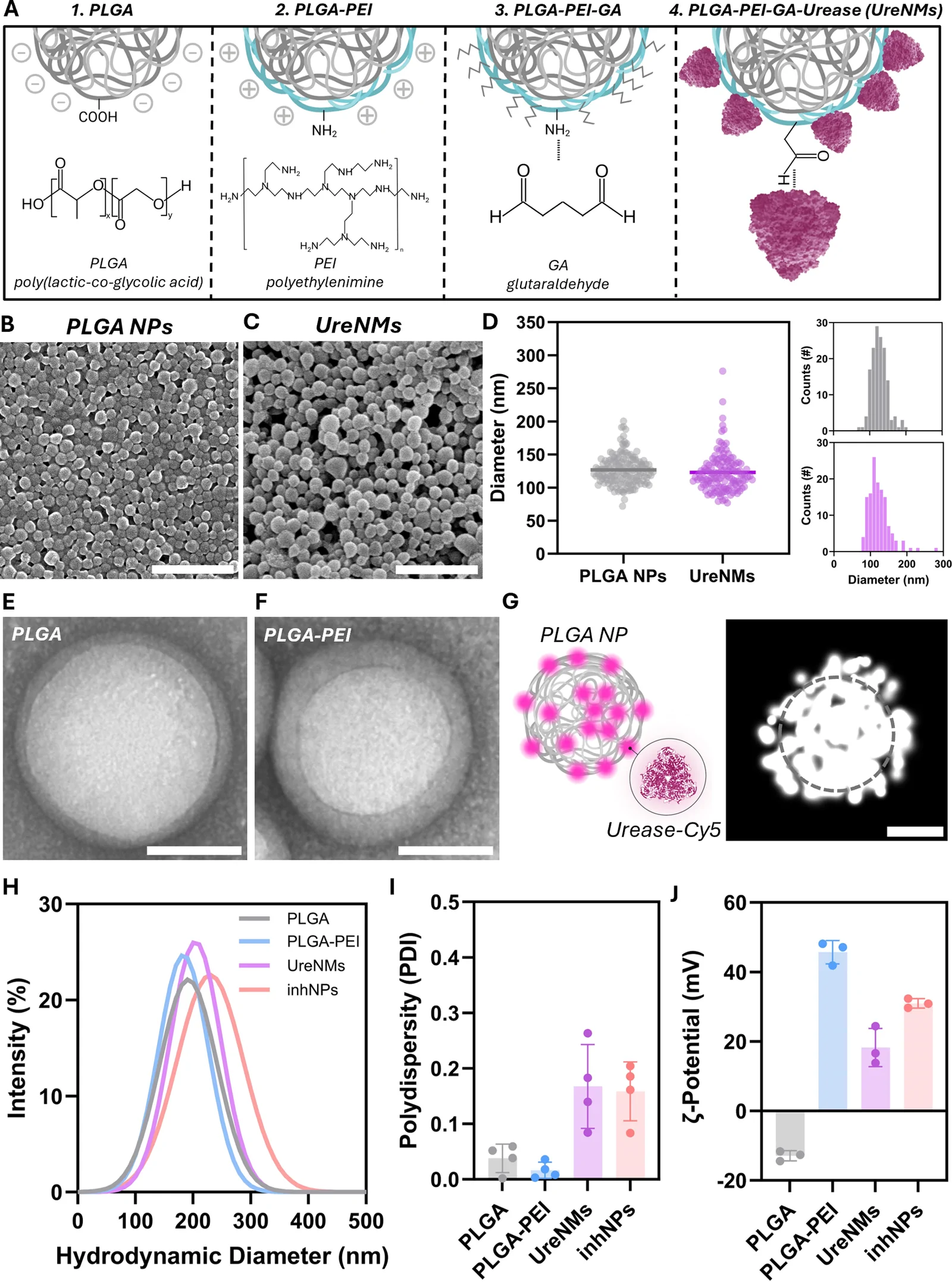
Urease-nanomotor synthesis and characterization. (**A**) Schematic of the functionalization process. From a PLGA nanoparticle (1), a positive layer of polyethylenimine is added (2) before using glutaraldehyde (3) to link urease (4). SEM was used to visualize PLGA NPs (**B**) and PLGA–PEI-urease (UreNMs) (**C**) (scale bar = 1000 nm). (**D**) The diameter measured by SEM was compared between PLGA NPs and UreNMs, and the size is represented as a distribution (top = PLGA NPs, bottom = UreNMs) (*n* = 150). TEM was used to obtain representative images of PLGA (**E**) and PLGA–PEI NPs (**F**) (scale bar = 50 nm). (**G**) Urease presence in UreNMs’ surface was visualized by STORM (scale bar = 100 nm) (represented as a sphere of 200 nm diameter with a dotted line). PLGA, PLGA–PEI, PLGA–PEI-urease (UreNMs), and PLGA–PEI-inhibited urease (inhNPs) were characterized by DLS to measure the hydrodynamic diameter distribution (Gaussian distribution) (**H**), the polydispersity (**I**), and the surface charge (**J**) (*n* = 4). Results are shown as mean ± SD. Created in BioRender. Prado Morales, C. (2026) https://BioRender.com/qdizc4h.

We used glutaraldehyde (GA) linking chemistry to generate a random asymmetric distribution of enzymes around the NPs, as previously reported.^27^ We covalently bound the amine groups provided by PEI with the primary amine groups of urease (present in the lateral chains of lysin and arginine). As a result, UreNMs were generated. The size and shape of UreNMs were visualized by SEM and TEM (in Figures 1C and S1H respectively), without observing a significant increase in the final mean diameter size (122.84 ± 29.10 nm) (Figure 1D). When UreNMs were analyzed by DLS, the hydrodynamic diameter resulted in 227.45 ± 16.74 nm (Figure S1E), an increase attributed to the clustering of the enzymes across the surface and the change in the surrounding hydration layer.^22^ DLS was further used to assess the success of the functionalization revealing a highly monodisperse NM population characterized by a single peak in the size distribution (Figure 1H) and PDI value lower than 0.2 (0.17 ± 0.08) (Figure 1I). The GA amount used for functionalizing the NPs was minimized to prevent a significant increase in PDI, in contrast to the aggregation observed in UreNMs functionalized with higher GA percentages (Figure S1I). Furthermore, the electrostatic repulsion arising from the positive surface charge of the resulting PLGA-PEI-GA NPs minimized the possible aggregation (Figure S1J), even in the absence of urease attached (Figure S1K).

The presence of urease on the surface of UreNMs was qualitatively visualized by stochastic optical reconstruction microscopy (STORM) (Figure 1G) and quantified by bicinchoninic acid (BCA) protein assay (66.78 ± 11.10 μg of urease/mg of PLGA).

In parallel, inactive control NMs were synthesized by chemically inhibiting urease (Figure S2). The resulting passive NPs (inhNPs) were used as a control lacking catalytic activity (Figure 1H–J). The enzymatic activity test performed on UreNMs and inhNPs in the absence (0 mM) and presence (100 mM) of urea corroborated that only active NMs were able to catalyze the decomposition of the substrate, producing ammonium (Figure 2A). We characterized UreNMs to obtain the kinetic constants after adjusting a Michaelis–Menten curve (Figure 2B) (eq 1, see Methods). Our active NMs showed a catalytic reaction maximum velocity of 29.62 ± 3.37 μM/s and a *K*_m_ of 6.904 ± 3.574 mM, comparable to the values previously reported for organic urease NMs.^22^

**Figure 2:**
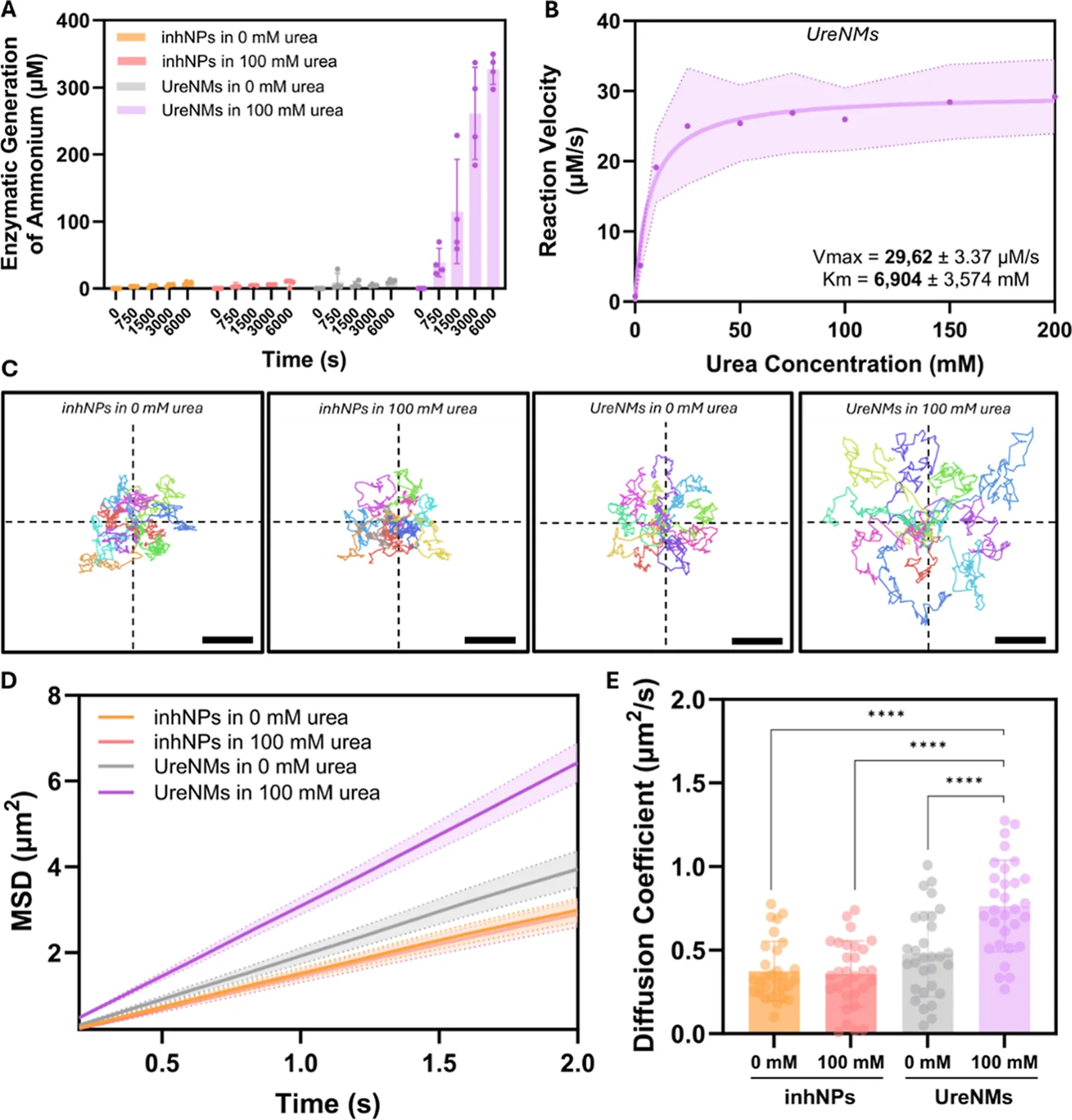
Urease-nanomotors enzymatic activity and movement at the single particle level. (**A**) Measurement of the ammonia generated by inhNPs and UreNMs in the absence (0 mM) and presence (100 mM) of urea over 6000 s (100 min). Values were normalized to time 0 s for each replicate (*n* = 4). Results are shown as mean ± SD. (**B**) Characterization of the kinetic parameters of UreNMs by representing the reaction velocity at different urea concentrations and fitting a Michaelis–Menten curve (solid line) (*n* = 4). Results are shown as mean ± SD. (**C**) Ten representative trajectories of individual inhNPs or UreNMs in the absence (0 mM) or presence (100 mM) of urea during 30 s of movement were plotted (scale bar = 3 μm). From each individual particle, the mean square displacement (MSD) (*n* = 30) (results are shown as mean ± SEM) (**D**) and the diffusion coefficient (**E**) (*n* = 30) (results are shown as mean ± SD) were calculated and plotted. ****p < 0.0001.

### Movement of UreNMs from Single Particle to Collective Motion

We evaluated the self-propulsion of individual UreNMs by recording fluorescent active NMs or passive inhNPs in the absence (0 mM) or presence (100 mM) of urea and representing their trajectories (Figure 2C). We observed an increased traveled distance of UreNMs when compared to the controls. To further visualize the active motion, the video-recorded trajectories were processed with a custom-made Python code to obtain the mean square displacement (MSD), as represented in Figure 2D. inhNPs either with or without urea and UreNMs in 0 mM urea show a linear diffusive behavior as expected for passive NPs undergoing Brownian motion.^53^ On the contrary, UreNMs in 100 mM urea showed significantly enhanced MSD values, characteristic of self-propelled NMs (Figures 2D and S3A).^29,54^ The diffusion coefficients for all the conditions were obtained after fitting the MSD curves with eq 2 (see Methods), being 0.77 ± 0.27 μm^2^/s for UreNMs in 100 mM urea (Figure 2E), which is 61% higher than the control in the absence of a substrate (Figure S3B). Active single particle motion of UreNMs can be explained by their unique ability to decompose urea and produce an asymmetric gradient of products around the NPs, enabling active ionic self-diffusiophoresis.^55,56^

We next investigated the collective behavior of UreNMs. Collective dynamics, or swarming behavior, of enzymatic NMs have been previously demonstrated and studied.^22,50,57–60^ A drop of highly concentrated Cy5-NMs was deployed in a solution containing urea at 0 and 100 mM and recorded at low magnification (1.25×) (Figure 3A and Videos S1, S2, and S3). A homemade Python code was used to calculate the covered area by the collective motion observed in the videos under each condition.^25^ UreNMs sedimented in the absence of urea and showed slow linear diffusion at the bottom of the plate, covering an area of 32.17 ± 7.02 mm^2^ after 120 s (Figure 3B). The control inhNPs in 100 mM urea showed the absence of sedimentation and an increased linear diffusion, covering 72.99 ± 4.41 mm^2^ after 120 s (Figure 3B). This phenomenon can be explained by the buoyancy effect, which has formerly been reported in relation to the swarming behavior of enzymatic NMs.^57^

**Figure 3:**
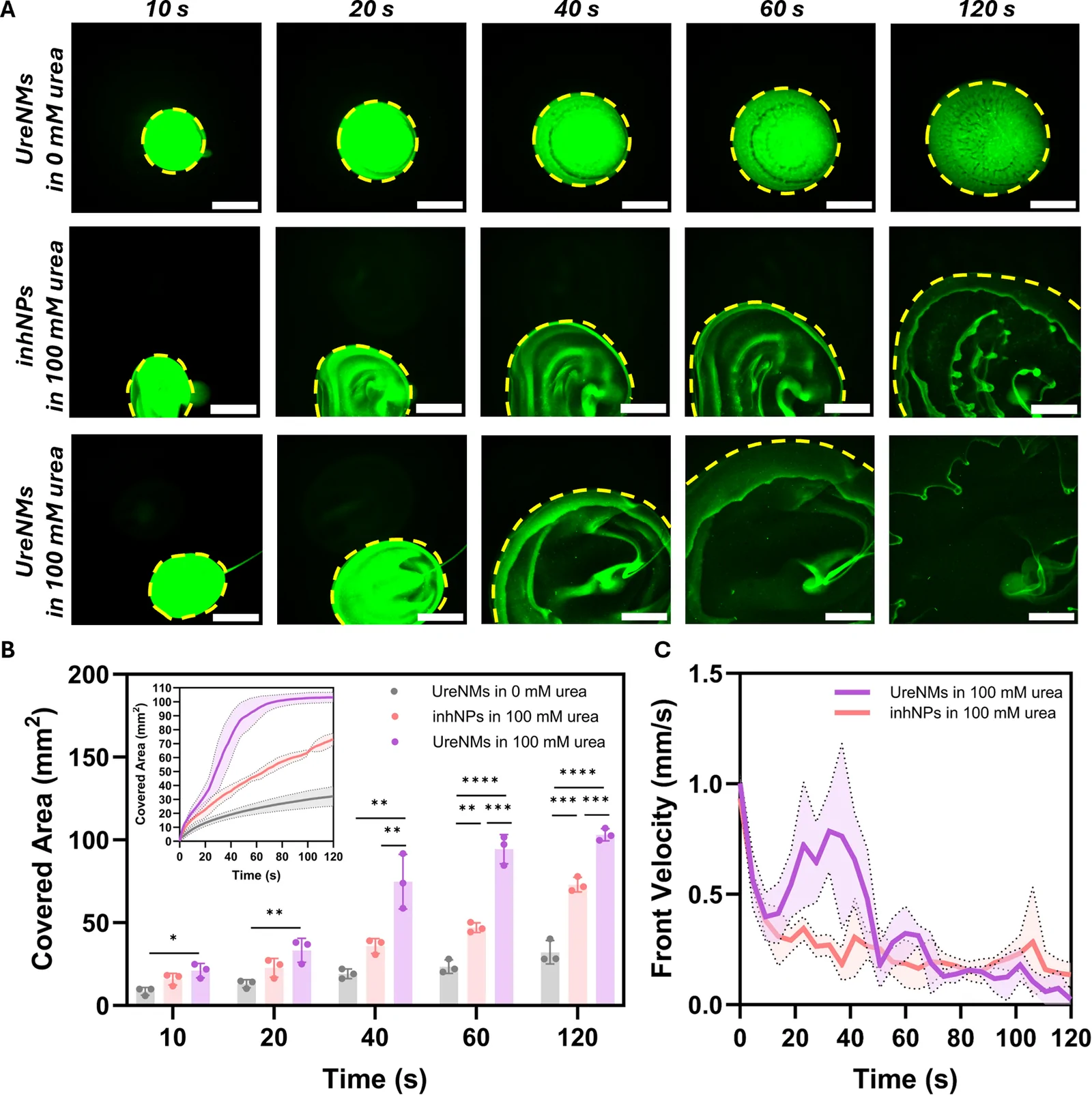
Collective motion of nanomotors. A drop of Cy5-labeled UreNMs and inhNPs was added to a dish with 0 or 100 mM urea and recorded for 120 s. (**A**) Representative videos were chosen, and screenshots at time points of 10, 20, 40, 60, and 120 s were depicted, with a yellow dotted line bordering the front of the swarm (scale bar = 2500 μm). (**B**) A homemade Python code was used to analyze the covered area over time (inlet), and the specific values at 10, 20, 40, 60, and 120 s were graphed and compared. (**C**) The same Python code was used to determine the front velocity of the swarm of UreNMs and inhNPs in 100 mM urea (*n* = 3). Results are shown as mean ± SD *p < 0.05, **p < 0.01, ***p < 0.001, ****p < 0.0001.

Here, we confirm that our system follows the same underlying principles previously reported by recording NM behavior from a side view (Figure S4A). When the density of the particulate (NPs + PBS droplet) is lower or equal to the surrounding density, it sediments, as observed for NMs in PBS. In contrast, when the surrounding medium contains 100 mM urea, its density slightly increases, resulting in an upward displacement of the particulate phase (Figure S4B,D,E,F,G). This behavior is dependent on urea concentration; when the medium contains 50 mM urea and is less dense, the upward velocity decreases, as depicted in Figure S4B, which was previously demonstrated both experimentally and through computational modeling.^57^ Importantly, the buoyancy effect is independent of the catalytic activity of the NMs, as both active UreNMs and inhNPs exhibit the same tendency: sedimenting in the absence of urea (0 mM) and moving upward at higher urea concentrations (100 mM). Nevertheless, when we image UreNMs in 100 mM urea from the top, we can see active swarming behavior and a coverage of the entire surface in 60 s (94.47 ± 3.69 mm^2^), a 2-fold larger area compared to inhNPs in 100 mM urea and 4-fold larger area than UreNMs in 0 mM urea at the same time point. After 20 s, the front of the NM swarm showed active acceleration and increased velocity, a phenomenon that is not observed in the inhNPs in urea (Figure 3C). We observed the same tendency of UreNMs in urea to cover larger longitudinal surfaces in the side view-recorded videos (Figure S4C). Altogether, although neither UreNMs nor inhNPs sediment when dispersed in 100 mM urea due to buoyancy, only active NMs can catalyze urea decomposition and actively expand, covering larger surface areas.

### In Vitro Degradation of PLGA NMs

We performed a degradation study comparing UreNMs with a standard solution of PLGA NPs. UreNMs and PLGA NPs were kept in aqueous solution at 37 °C under constant shaking to ensure that they are only exposed to hydrolysis. Literature suggests that PLGA NP degradation occurs due to the random hydrolytic chain scission of the ester bonds connecting both monomers, lactic acid, and glycolic acid.^61,62^ We analyzed weekly each sample by SEM, spectrophotometry for optical density (OD), and DLS over a 15 week period. UreNMs maintain the spherical uniform shape and size during the first weeks of incubation (Figures 4A,B and S5A). Our data align with the reported bulk degradation mechanism of PLGA NPs at the initial stage, in which the molecular weights of the polymers decrease without significantly affecting the total mass.^61^ From week 7 onward, we observe an initial decrease in size, which corresponds to the second phase of the mechanism, during which it is reported that the mass is significantly reduced (Figures 4A,B and S5A).^61^ To validate the structural alteration of the NPs’ chassis, we used SAXS for analyzing both designs after 13 weeks of incubation.^63^ Figure 4C,D demonstrates an evident decrease in intensity of the scattering profiles when compared to freshly prepared PLGA NPs and UreNMs, estimated as the scattering invariant (Q) (Figure 4E). This reduction suggests a decrease in scattering contrast, attributed to the development of a less compact internal structure and increased surface roughness. These changes are consistent with structural corrosion and swelling processes expected from this stage of PLGA NP degradation. Both PLGA NPs and UreNMs showed similar scattering invariant (Q) reductions (57.30% ± 9.56 and 50.81% ± 8.37, respectively), evidencing comparable degradation rates.

**Figure 4:**
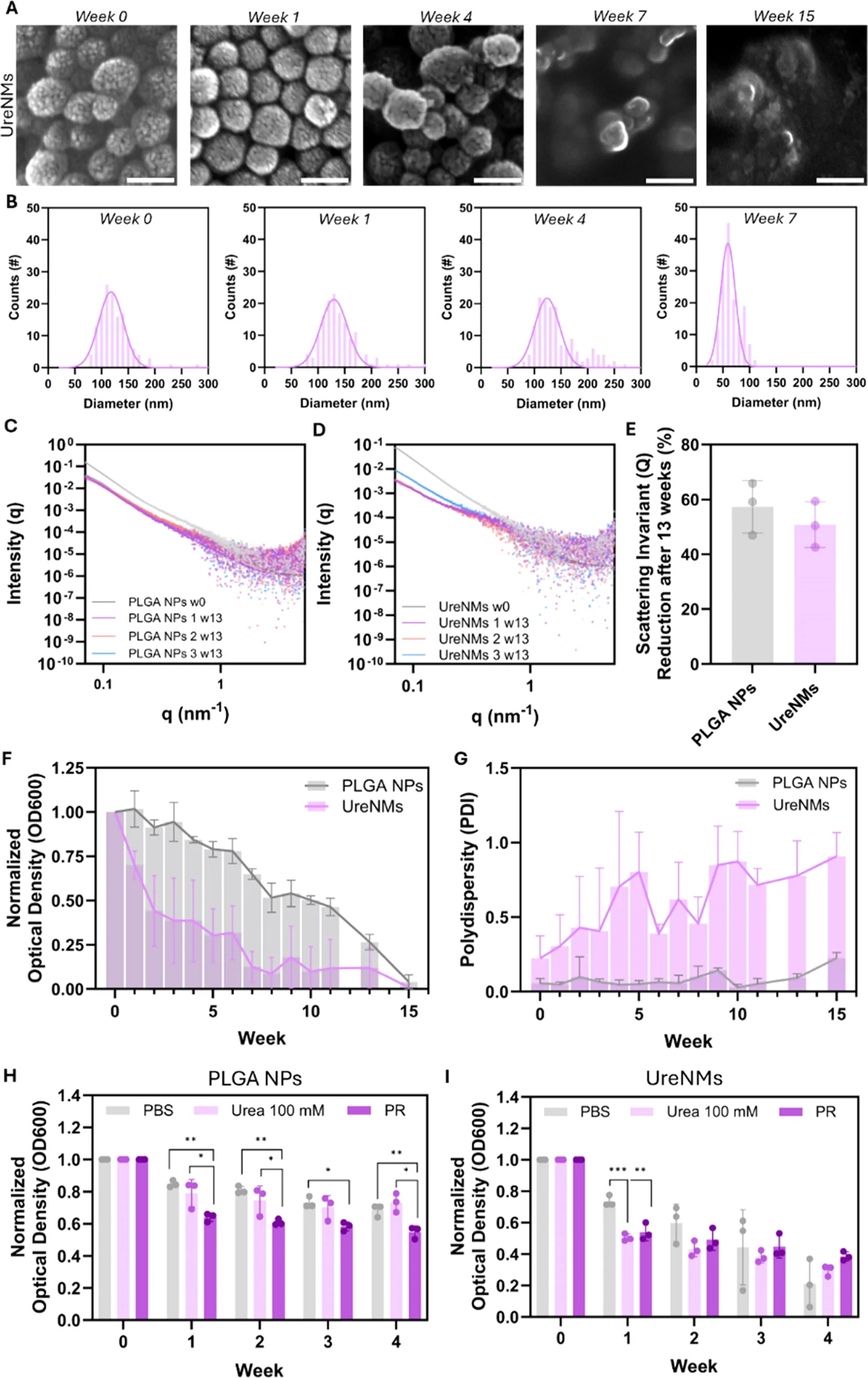
Nanomotors degradation. (**A**) UreNMs in water were kept at 37 °C, and their morphology was checked by SEM every week (scale bar = 150 nm) (*n* = 3). (**B**) SEM images were further used to quantify the diameter of UreNMs throughout the weeks and represent them as a Gaussian distribution (line) [*n* = 150 (50 measurements/replicate)]. SAXS was used to measure the scattering profile of PLGA NPs (**C**) and UreNMs (**D**) freshly prepared (week 0) and after 13 weeks of incubation in PBS at 37 °C. (**E**) SAXS data were used to extract the scattering invariant (Q), and they were represented as the reduction percentage compared to the value obtained at week 0. (**F**) The optical density at 600 nm (OD600) of UreNMs and PLGA NPs was measured as a direct way of quantifying their concentration over the different weeks, and the values were normalized to the week 0 OD600 for each replicate (*n* = 3). (**G**) At the same time points, DLS was used to follow the change in the polydispersity index of UreNMs and PLGA NPs (*n* = 3). The same experiment of measuring OD600 was used to study the degradation of PLGA NPs (**H**) and UreNMs (**I**) in 100 mM urea and the products of the reaction (PR). Results are shown as mean ± SD *p < 0.05, **p < 0.01, ***p < 0.001.

By week 15, we observe major degradation as spherical shapes were no longer visible, and UreNMs could not be observed (Figures 4A and S5A). The OD at 600 nm (OD600) was used as a direct way of measuring the changes in the concentration of our sample solutions (Figure S5B,C). For both UreNMs and PLGA NPs, a gradual decrease in OD600 was observed over the weeks, reaching very low values after week 15 (Figure 4F).This trend was further supported by a visible reduction in UreNM concentration (Figure S5A). Our empirical results are consistent with the literature where researchers observed a complete degradation of PLGA particles after 14–15 weeks and between 12 and 24 weeks depending on the composition and size.^64,65^ The faster decrease in OD600 observed for UreNMs compared to PLGA NPs (Figure 4F) can be attributed, at least in part, to the increased PDI values observed in Figure 4G. We hypothesize that as the PLGA chassis of the UreNMs degrades, the remaining components of the design may disperse and tend to increase aggregation, resulting in an increase in the PDI (Figure 4G). However, we expect similar degradation kinetics for both designs, as indicated by the SAXS data previously discussed (Figure 4E). Overall, our studies confirm that UreNMs undergo PLGA-like hydrolytic degradation with near-complete decomposition observed after 15 weeks.

Once we proved the degradability of UreNMs under aqueous conditions, we investigated the effect of urea in the degradation rate. UreNMs incubated in 100 mM urea exhibited a decrease in the OD of the solution compared to the PBS control, whereas the OD of PLGA NPs remained unaffected (Figure 4H,I). This enhanced degradation of the active UreNMs in the presence of urea is attributed to the increase in pH resulting from the catalytic reaction. When PLGA NPs were incubated with the products of the reaction (PR), which exhibited a pH of approximately 9 (Figure S11B), the OD also decreased (Figure 4H). In the case of UreNMs, we observed a similar decay of OD for urea at 100 mM and the PR (Figure 4I). These findings indicate that PR promotes the degradation of both UreNMs and PLGA NPs, whereas urea alone is ineffective. Our results are consistent with previous reports demonstrating accelerated PLGA hydrolysis under alkaline conditions.^66,67^

### Biocompatibility of Human Fibroblasts in a 3D Collagen Matrix (Dermis-like) with UreNMs

Ensuring biocompatibility is fundamental in the development of new NP platforms. We assessed the biocompatibility of UreNMs in a dermis-like 3D model. The model was based on human-derived fibroblasts (HDF) cultured in a 3D collagen matrix, providing a more relevant model compared to traditional 2D cell cultures (Figure 5A).^41^ We visualized collagen fiber disposition with a label-free confocal strategy, using refractive microscopy (Figure 5B). In addition, upon incubation, we observed Cy5-UreNMs between the collagen fibers and the HDFs (Figure 5C). Cells looked phenotypically healthy and unaffected after incubation with UreNMs at 50 μg/mL and 100 mM urea. We used the CellTiter-Glo 3D cell viability assay to quantify the cytotoxicity of NMs by measuring ATP levels as a viability indicator. The models were incubated with inhNPs and UreNMs with 0 mM and 100 mM urea for 1 h. Afterward, we removed the nonpenetrated NPs and kept the cells for 24 h to assess the resulting effects. High doses (150 μg/mL) of inhNPs or UreNMs, as well as 100 mM urea alone, did not affect the viability of the cells. Products of the reaction generated by the decomposition of urea by UreNMs showed a significant increase in cytotoxicity at 150 μg/mL but a safe profile when using lower doses such as 50 μg/mL (Figure 5D,E).

**Figure 5:**
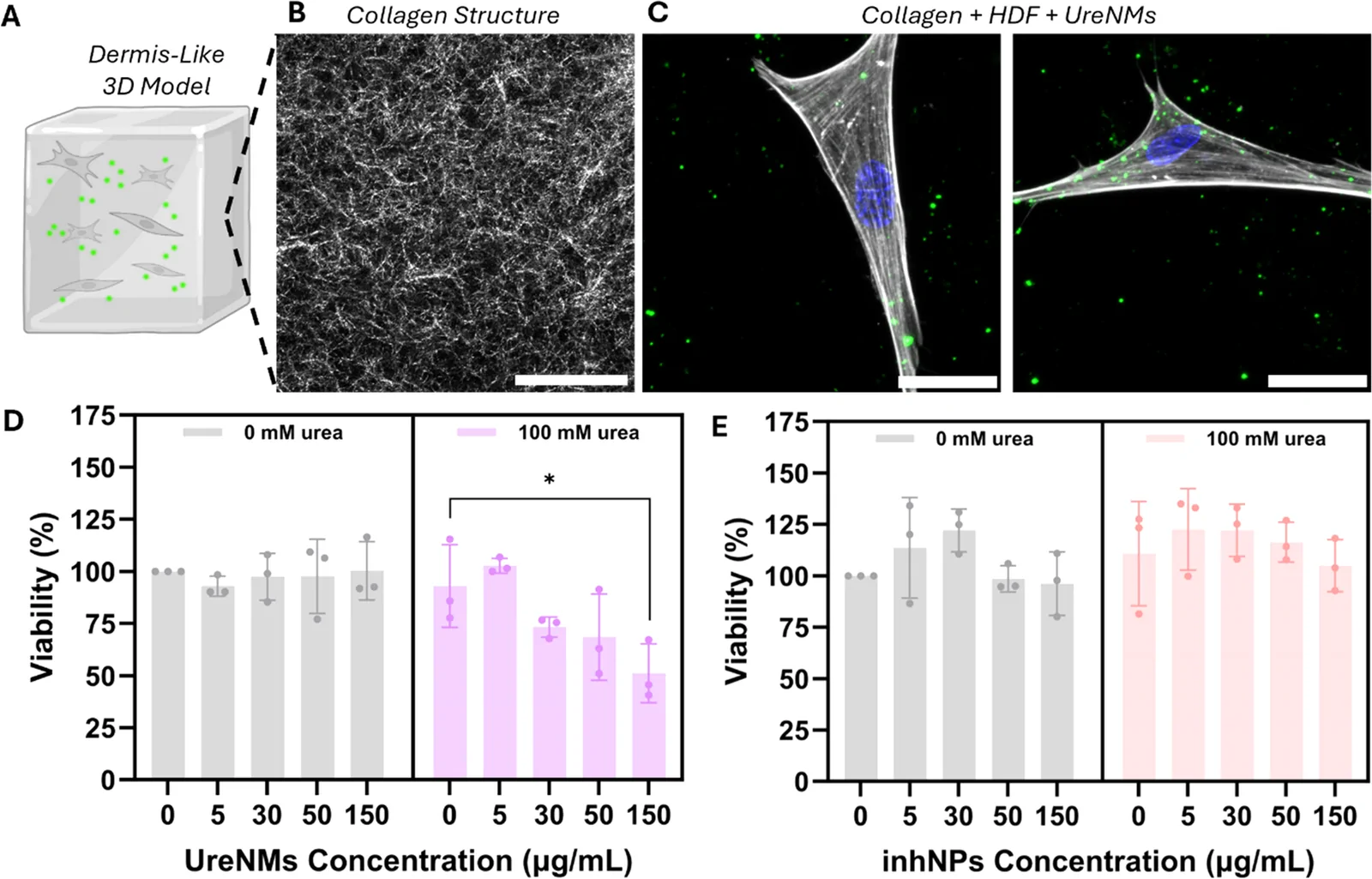
Biocompatibility of UreNMs in a 3D dermis model. (**A**) Scheme of the 3D collagen matrix with human-derived fibroblasts (HDF) used to mimic the human dermis. (**B**) Refraction confocal microscopy excited at 648 nm was used to visualize the collagen network (scale bar = 50 μm). (**C**) Cy5-UreNMs (green) at 50 μg/mL, and 100 mM urea was added to the model and incubated for 1 h before removal and 24 h of extra culture. Afterward, fixed models were stained with ActinRed-555 (HDFs’ actin labeling, gray) and Hoechst (HDFs’ nuclei labeling, blue) and imaged in 3D with a Z-stack (scale bar = 30 μm). To quantify the biocompatibility of UreNMs (**D**) and inhNPs (**E**) at different concentrations (0, 5, 30, 50, and 150 μg/mL) in the absence (0 mM) and presence (100 mM) of urea, CellTiter-Glo 3D viability assay was used to measure the ATP produced by cells in each condition after 1 + 24 h incubation (*n* = 3). Results are shown as mean ± SD *p < 0.05. Created in BioRender. Prado Morales, C. (2026) https://BioRender.com/lay7phs.

### Development and Characterization of a Reconstructed Human Skin Model

To evaluate the crossing capabilities of UreNMs through skin layers, we developed a model that better mimics the epidermal architecture and barrier function of human skin compared with previously used murine models.^33–36^ We generated human skin equivalents, as depicted in Figure 6A, where the dermal fibroblasts and keratinocytes (KCs) used were isolated from human skin donors. The dermal matrix was produced by embedding fibroblasts in collagen, while KCs were seeded on top to establish the basal epidermal layer. Subsequent culture at the air–liquid interface (ALI) in a supplemented medium promoted KC differentiation and the formation of stratified epidermal layers, ultimately leading to the development of the SC (Figure 6B). We validated the proper organization and differentiation of the epidermis in our models by assessing the expression of early (K15), intermediate (K10), and late (Loricrin) differentiation markers and comparing them to those observed in native human skin (Figure 6B).^68^ To address interdonor variability in skin formation, we generated three biological replicates using cells from three independent donors. We observed no differences in tissue architecture (H&E) or in epidermal differentiation marker expression (K15, K10, and loricrin) (Figures 6B and S6).

**Figure 6:**
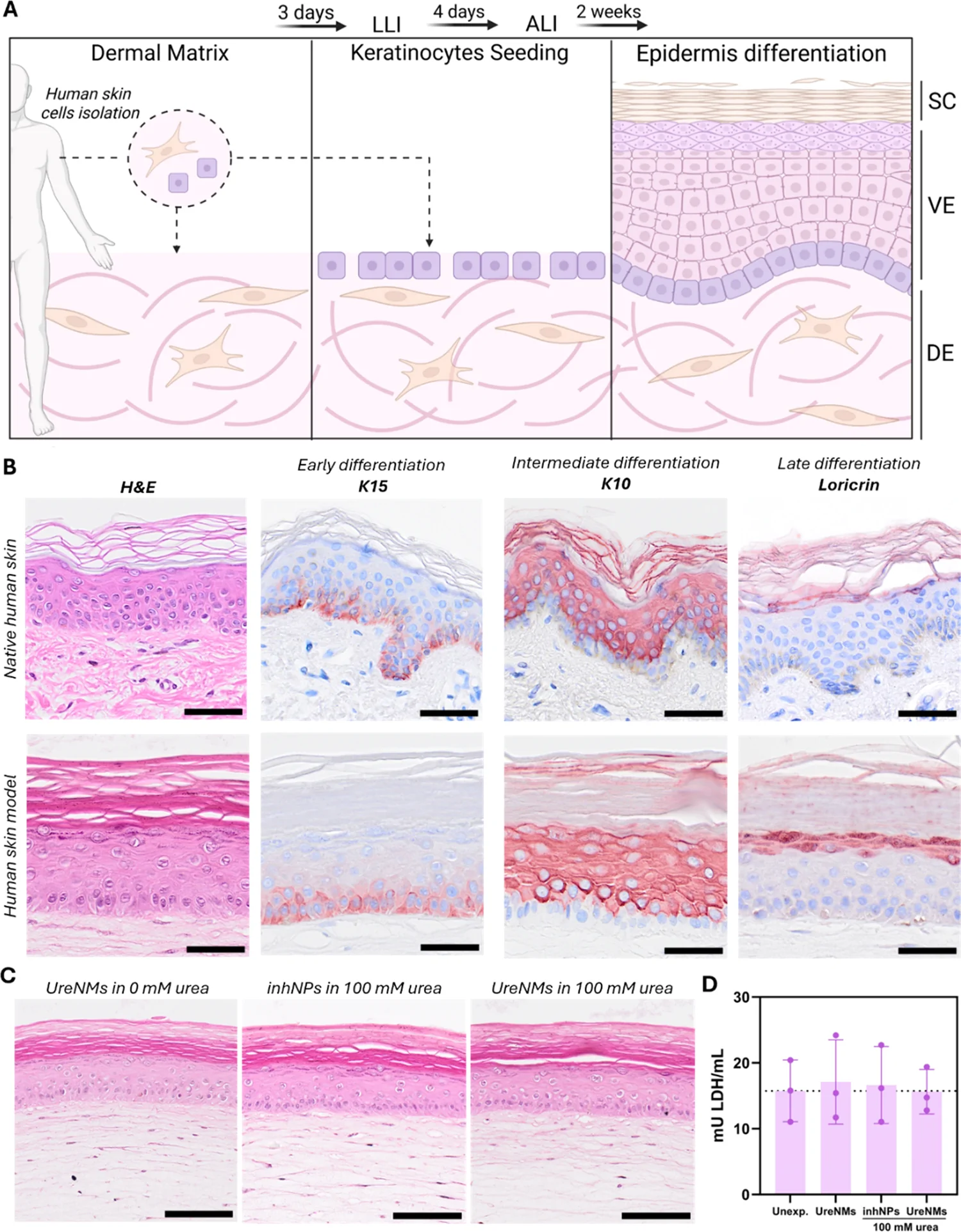
Human skin model generation, characterization, and nanomotor compatibility studies. (**A**) A summary of the main steps followed to produce a human skin model in vitro was represented (LLI = liquid–liquid interface, ALI = air–liquid interface, SC = stratum corneum, VE = viable epidermis, DE = dermis). (**B**) Skin architecture and epidermal differentiation were visualized by H&E and immunohistochemistry against keratin 15 (K15, as a basal layer marker), keratin 10 (K10, as a suprabasal epidermal differentiation marker), and Loricrin (as a terminal differentiation marker) (scale bar = 50 μm). UreNMs and inhNPs at 300 μg/mL with 0 mM and 100 mM urea were added to the top of the skin models and incubated for 24 h. Then, effects on the skin architecture (**C**) and viability (**D**) were checked by H&E staining and LDH supernatant quantification, respectively (scale bar = 100 μm) (*n* = 3). Results are shown as mean ± SD. Created in BioRender. Prado Morales, C. (2026) https://BioRender.com/ljwbcaf.

We assessed the safety of UreNMs by observing the skin architecture and performing a cytotoxicity assay after incubation. We topically applied a drop of sterile UreNMs and inhNPs at 300 μg/mL in 0 mM and 100 mM urea onto the human skin models previously reconstructed in vitro. Histological analysis showed no macroscopic alterations in the dermal and epidermal architecture when the models were incubated with UreNMs and urea (Figures 6C and S7), in contrast to the physical disruption of the skin observed with other transdermal enhancement techniques.^69–71^ Further assessment of skin integrity was performed by quantifying the amount of lactate dehydrogenase (LDH) released from damaged cells into the medium, providing a direct measure of tissue viability.^68^ The literature demonstrates that the topical exposure to damaging agents does result in increased LDH release, indicating that skin models are indeed capable of producing LDH in response to cytotoxic stimuli.^72^ As shown in Figure 6D, no increase in LDH levels was detected after incubation with UreNMs in 100 mM urea, proving that the tissue viability was not affected. Overall, UreNMs were demonstrated to be safe, preserving both skin macroscopic architecture and tissue viability.

### UreNMs Crossing Human Skin Models

Most NP formulations are unable to overcome the skin barrier via transepidermal routes, accumulating primarily in hair follicles, which represents a negligible entry pathway for therapeutic purposes. In this study, we assessed the penetration of UreNMs by using a reconstructed human skin (RhS) model in vitro. This model allows us to evaluate the crossing capabilities in intact, healthy skin while excluding the interference from transappendageal routes, thereby ensuring assessment of transepidermal transport. We generated 2–3 independent skin equivalent models (technical replicates) from 3 distinct human donors (biological replicates) (Scheme 1). Then, we topically applied Cy5-UreNMs in the absence and presence of urea, as well as the control Cy5-inhNPs with urea, onto the human skin models and incubated them for 24 h. The architecture of the skin can be distinguished by the different cellular densities across its layers, a feature we used to define three regions of interest (ROIs): dermis (DE), viable epidermis (VE), and SC. In the DE region, cells are sparsely distributed, resulting in a low overall cellular density. In contrast, the VE is composed of several layers of tightly packed KCs, leading to a high-cell-density region. Finally, the SC is composed of dead KCs and does not exhibit cellular density with nuclei staining (Scheme 1 and Figure 7A).^41^

**Scheme 1:**
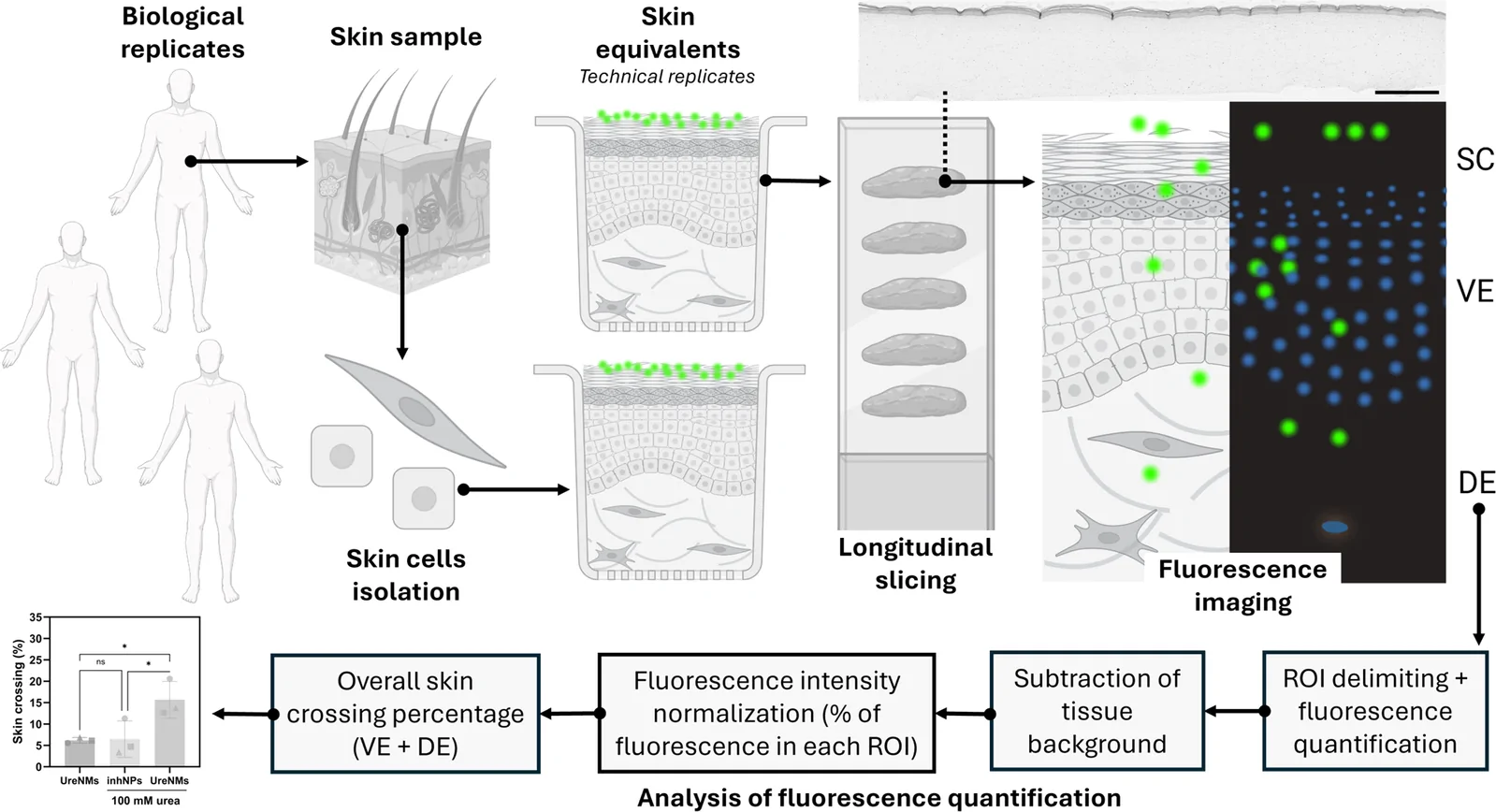
Human Skin Crossing Studies Setup^a^

a Scheme of the different steps followed during skin crossing experiments. Skin samples from each human donor (biological replicate) were obtained to isolate KCs and dermal fibroblasts. These pools of cells were used to generate 2–3 models (technical replicates) from each biological replicate. After NM incubation, each tissue was sliced along the model and imaged with fluorescence microscopy. Complete longitudinal tissue images were used to quantify the fluorescence (NPs) signal coming from the three delimited ROIs (stratum corneum, viable epidermis, and dermis). Each ROI was delimited depending on the characteristic cellular density. Unexposed samples were used as a tissue background, and it was subtracted from each sample. Then, the fluorescence intensity was normalized as a percentage of localization in each ROI, considering that the total fluorescence in each image is 100%. Finally, the crossing percentage was calculated as the presence in viable epidermis and dermis, and it was plotted. Created in BioRender. Prado Morales, C. (2026) https://BioRender.com/5huvl48.

**Figure 7:**
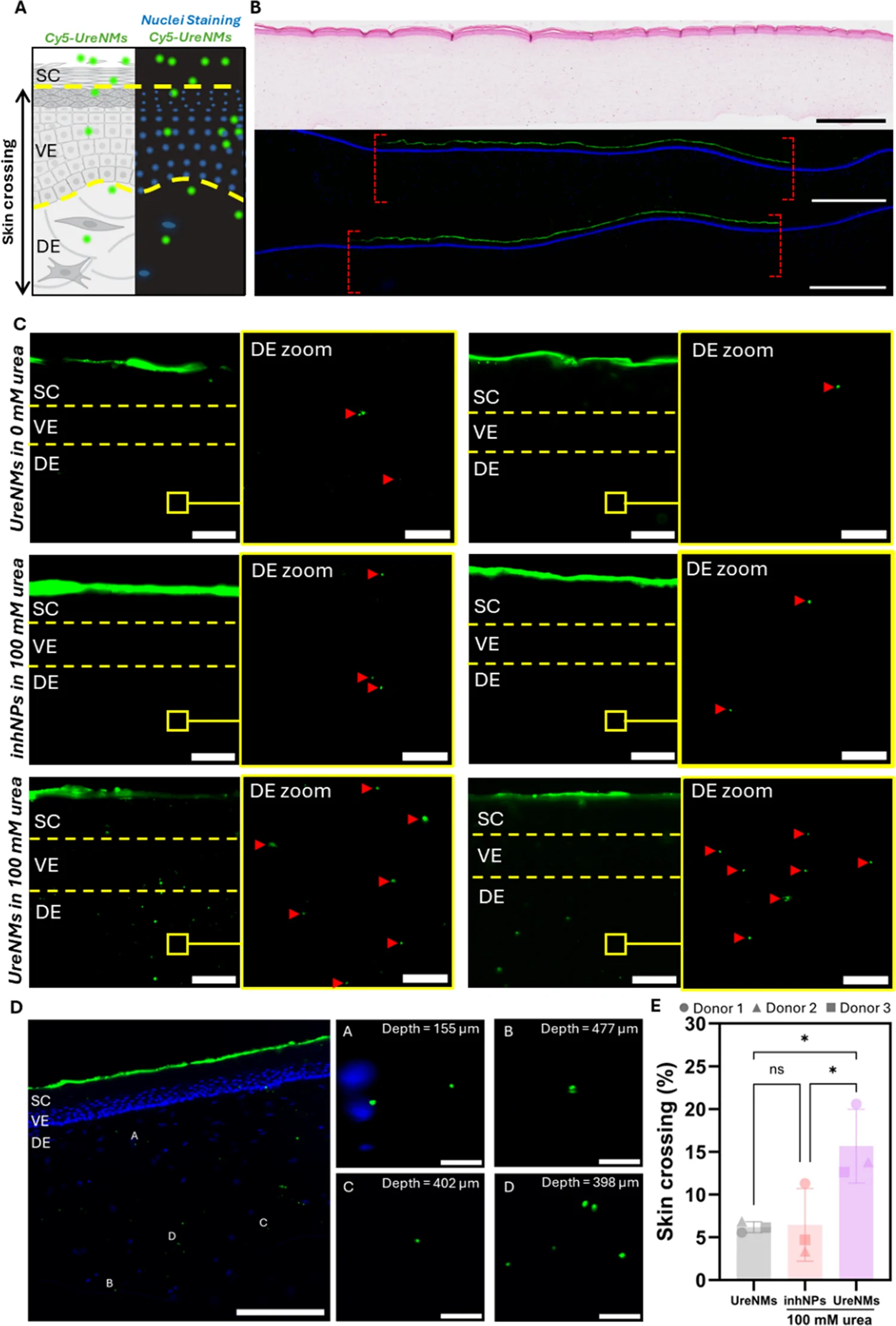
Nanomotors crossing human skin models. (**A**) Representation of the method followed to quantify the amount of particles in each region of interest (ROI): stratum corneum (SC), viable epidermis (VE), and dermis (DE). Cy5-labeled UreNMs and inhNPs (in green) were topically applied in the absence (0 mM) and presence (100 mM) of urea for 24 h, and skin samples were sectioned and stained with DAPI (nuclear labeling, blue). (**B**) Complete longitudinal slide images were used to ensure the lack of leakage through the borders and to limit the analysis area between the red lines (scale bar = 1000 μm). (**C**) Fluorescence images of the skin sections were taken, and yellow dotted lines were used to limit the 3 ROIs (scale bar = 50 μm). Then, zoomed-in images of the dermis were obtained, and the NPs detected were indicated with red arrows (scale bar = 15 μm). (**D**) Zoomed-out images were also acquired to visualize the penetration depth of UreNMs (scale bar left image = 200 μm, scale bar right images = 15 μm). (**E**) From the pictures and values obtained, the skin crossing percentage was calculated in the different conditions as the combination of the NPs presence in the dermis and viable epidermis (*n* = 3). Results are shown as mean ± SD *p < 0.05. Created in BioRender. Prado Morales, C. (2026) https://BioRender.com/i7a38ql.

An average of 5 complete longitudinal sections was analyzed for each technical replicate to quantify the presence of Cy5-UreNMs within the 3 predefined ROIs. ROIs were manually defined based on cellular densities, determined by DAPI staining, within the SC, VE, and DE, as depicted in Figures 7A and S8A. We used complete longitudinal tissue images, like those in Figure 7B, to confirm that leakage through the model borders did not occur and to quantify the NMs crossing large representative skin areas. Images in Figures 7C and S9 demonstrate that UreNMs and inhNPs primarily accumulate in the SC, with penetration into the VE and DE occurring only when UreNMs are combined with 100 mM urea. Furthermore, UreNMs that successfully traverse the SC can subsequently diffuse and extend throughout the dermis compartment (Figure 7D). To quantify the NM localization, total fluorescence intensity was measured within each ROI in the complete longitudinal slices. We corrected our measurements by subtracting the tissue background measured in each specific ROI from the unexposed samples. For each technical replicate, all images were averaged and expressed as percentage distribution across ROIs, setting 100% to the sum of fluorescence measured in SC, VE, and DE. Finally, we averaged the technical replicates to obtain the overall percentage of NM localization in each region (Scheme 1).

The skin crossing percentage was calculated based on the amount of NPs detected within the VE and DE (Figure 7A). Results indicate that UreNMs in the presence of 100 mM urea overcome the SC significantly more effectively than the same UreNMs in the absence of the fuel, reaching a skin crossing percentage of 15.67 ± 4.30%, compared with 6.18% for the control (Figure 7E). Based on an initial UreNM concentration of 300 μg/mL, we estimated that a maximum of 50 μg/mL of UreNMs reaches the inner skin layers, an amount considered to be within a safe range, as shown in Figure 5D.

Urea is widely used and well tolerated in dermatological and cosmetic formulations due to its versatile properties.^51,73^ At lower concentrations (2–10% = 333–1665 mM), urea exhibits a hydrating effect, helping to maintain healthy skin and the protective barrier. In contrast, higher concentrations ranging from 10% (1665 mM) to 50% (8.3 M) are used as topical drug enhancers due to their keratolytic action.^51,73,74^ To further confirm that the urea concentration used in our experiments (100 mM = 0.6%) had no effect as a transdermal enhancer, we included the passive control of inhNPs. When inhNPs are topically administered with 100 mM urea, the skin crossing percentage shows no significant differences from the UreNMs control in the absence of fuel (Figure 7E). The results confirm that urea itself is not responsible for the enhanced skin penetration observed with UreNMs in combination with the urea. On the basis of the crossing rate displayed in Figure S8C, active UreNMs with urea are twice as abundant in the viable epidermis and three times more abundant in the dermis compared to both passive controls. Overall, skin crossing is enhanced by 2.5-fold with urease-powered NMs in the presence of urea (Figure S8C).

To further validate our results, we used skin models that were supplemented with a lipid mix of palmitic acid, linoleic acid, arachidonic acid, and vitamin E and vitamin C during the epidermal differentiation. We hypothesized that the supplementation would enhance the lipidic deposition during epidermal stratification, thus enhancing the skin barrier function of the model. The immunohistochemistry characterization did not show visible changes in the epidermal stratification when compared with the nonsupplemented model (Figure S10A). Upon analysis of skin crossing, as previously done in Figure 7 (Scheme 1), we showed the same crossing tendency as previously described, with a crossing percentage of 16.99 ± 2.26% (mean ± SD) in the case of UreNMs in 100 mM urea (Figure S10B–D).

Among all types of NPs, only deformable vesicles have been shown to penetrate the skin through transepidermal routes. However, these systems are unstable, costly, and complex to manufacture and suffer from poor batch-to-batch reproducibility and limited drug-loading capabilities.^9,19,75^ In contrast, here, we demonstrated the use of a conventional organic NP (PLGA), a well-established and accepted drug delivery platform,^44,45^ to enhance skin penetration when it was modified into a NM. UreNMs are capable of crossing intact, healthy skin without the need for a physical disruption. Furthermore, their performance has been validated in a human-relevant model, significantly strengthening their translational potential.^37–39^

### Skin Crossing Mechanism of UreNMs

We next determined their skin crossing mechanism. Architecture and composition of SC play a primary role in the barrier function of skin.^76^ Flattened dead corneocytes act as spacers in a surrounding hydrophobic lipid matrix based on ceramides, free fatty acids, and cholesterol.^77^ Under physiological conditions, lipids of the SC present a lamellar ordered or solid phase organization, which hinders the passage of water and most chemical compounds.^78–80^ It has been reported that transdermal delivery can be enhanced by modulating intercellular lipid interactions and inducing disorganization within the lamellar membrane structures, so-called chemical enhancers.^74,81,82^ Commonly studied enhancers include solvents such as ethanol, terpenes such as menthol, and surfactants such as sodium dodecyl sulfate (SDS).^3,12^ Their macroscopic alteration of the lipid organization of the SC has been extensively investigated using SAXS and electron microscopy, among other techniques.^12,83^

In our case, to investigate the interaction of UreNMs within the lipidic lamellar structures of the SC, we developed a unilamellar lipid model based on 40% ceramide 3, 25% palmitic acid, 25% cholesterol, and 10% sulfate cholesterol (Figure 8A).^84^ This composition has been previously reported to mimic the SC lipid composition and study its lamellar organization.^85^ The resulting monodisperse solution of SC lipid models (Figure S11A) was incubated with inhNPs and UreNMs in the absence and presence of 100 mM urea. To evaluate the effect of UreNMs on the lipid organization of the vesicles, we used synchrotron radiation SAXS to obtain the scattering profile of the SC lipid models (Figure 8A). SC models incubated with inhNPs in the absence and presence of urea exhibited overlapping plots (Figure 8B). In contrast, incubation with UreNMs resulted in a clear change in the scattering profile when comparing conditions with and without urea (Figure 8D), evidencing that only active NMs with the fuel induce changes in the lipid organization of the model.

**Figure 8:**
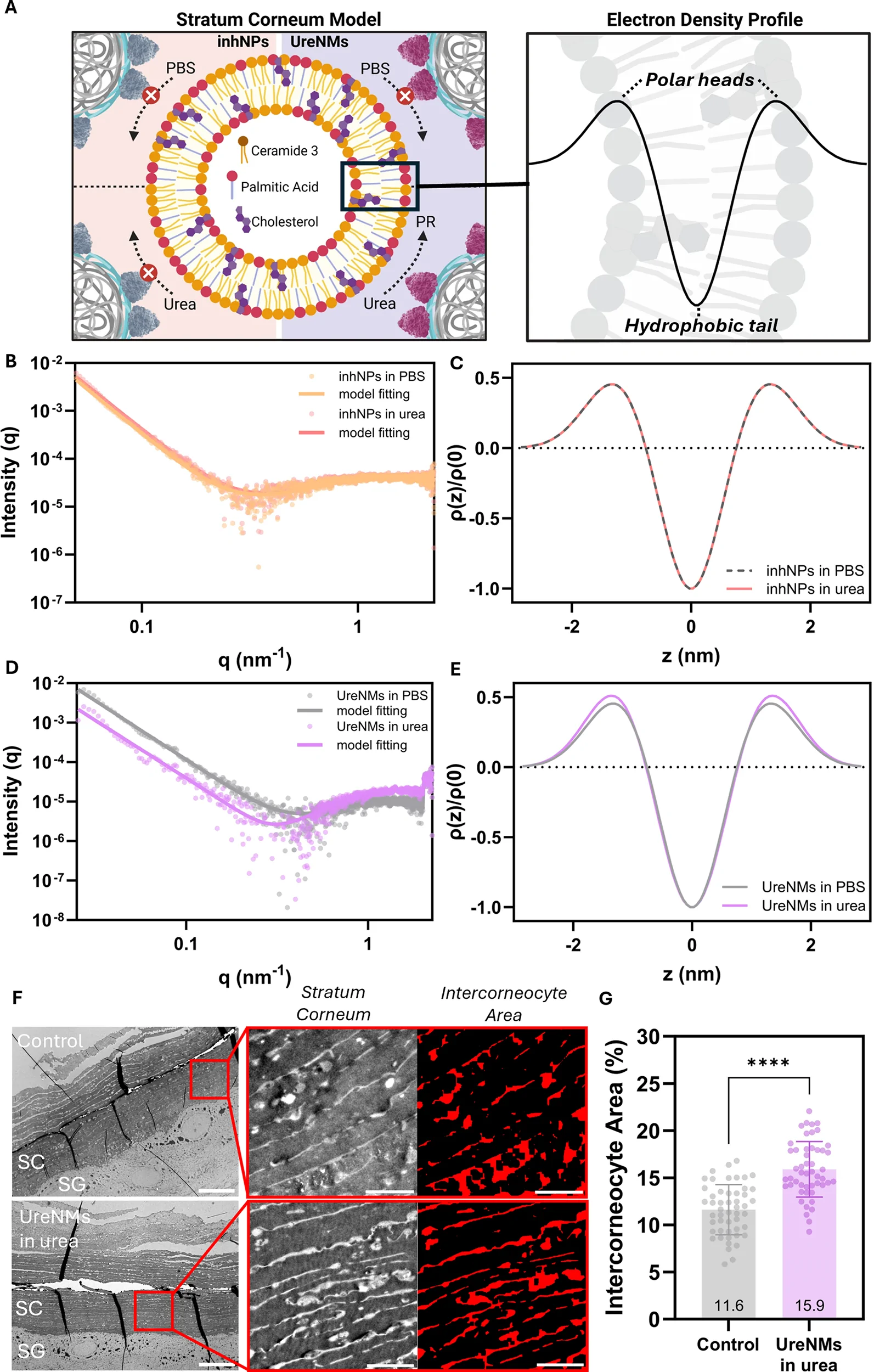
Mechanism of crossing. (**A**) A lipid model mimicking SC lipidic composition was generated and incubated with UreNMs or inhNPs in the absence or presence of urea. Changes in the membrane structure were analyzed by synchrotron radiation SAXS by transforming the scattering plots into electron density profiles. The scattering plots and electron density profiles were represented for inhNPs (**B**,**C**) and UreNMs (**D**,**E**). (**F**) Electron microscopy was used to visualize the effect of UreNMs with urea in the SC and compared with an unexposed control (left images scale bar = 10 μm, right images scale bar = 2 μm). (**G**) Image analysis was performed to quantify the intercorneocyte area in each condition (SC = stratum corneum, SG = stratum granulosum) (*n* = 1; 50 areas of SC were analyzed from 1 donor sample). Results are shown as mean ± SD ****p < 0.0001. Created in BioRender. Prado Morales, C. (2026) https://BioRender.com/8hoyibm.

To further investigate these changes, the scattering plots were converted into electron density profiles using a Fourier transform described in eq 4 (see Methods) and modeled as 3 Gaussian distributions (eq 5, see Methods), as shown in Figure 8A.^86,87^ The electron density distributions of SC lipid vesicles have been reported to closely replicate the molecular arrangement of native tissue.^88,89^ As expected, when inhNPs were incubated with urea, the electron density profile remained unchanged and overlapped with the one obtained with the nonfuel control (Figure 8C). Nonetheless, UreNMs in urea showed an increase in the electron density profiles of the polar heads when compared to the control without urea (Figure 8E). This change in the electron distribution across the membrane may result from a rearrangement of the lipid organization induced by UreNMs and their catalytic products.

The epidermis exhibits a pH gradient, with acidic values (∼5) at the SC that gradually increase toward neutrality, reaching physiological pH levels (∼7.4) in the basal layer.^90^ Several studies have confirmed the importance of epidermal pH in maintaining skin integrity and have shown that exposure to alkaline solutions induces rearrangements of the lipids in the lamellar bilayers, resulting in a more cohesive and rigid structure.^91–93^ These alterations align with our findings, which showed changes in the electron density profile of the polar head groups, likely arising from changes in the lipid organization induced by the pH increase to 9.24 caused by the catalytic activity of UreNMs (Figure S11B). To further validate our hypothesis, the same behavior was observed when SC models were incubated only with the products of the reaction (PR) catalyzed by free urease in the presence of urea (Figure S10C,D).

To corroborate our hypothesis, we aimed to assess the macroscopic effects of these SC lipid order alterations by imaging skin models incubated with UreNMs and urea using electron microscopy.^83^ This approach enabled the visualization of structural alterations in the SC, above the stratum granulosum (SG), at the submicron scale.^83^ Compared with unexposed controls, skin models incubated with UreNMs and urea exhibited a higher prevalence of imperfections within the lacunar SC network (Figure 8F), a feature previously associated with enhanced skin permeation.^83^ For quantitative assessment, 50 distinct SC regions were analyzed to determine the intercorneocyte area percentage. Incubation with UreNMs resulted in increased space between corneocytes from 11.62% ± 2.67 in unexposed controls to 15.90% ± 2.95 (Figure 8G) in UreNM-exposed samples. These results are consistent with our SAXS data and explain how UreNMs can overcome the SC by modulating its lamellar structures, an effect comparable to that reported for other skin chemical enhancers.^12,83,94^

Although the skin-crossing mechanism is shared between classical chemical enhancers and our technology, UreNMs offer the distinct advantage of acting as an NP-based drug delivery system. Meanwhile, classical enhancers face significant developmental limitations, require specific optimization for each API, and are limited to small drugs.^3,9^ UreNMs are a versatile platform with the potential to deliver a wide range of cargos and therapeutic agents.^28,50,95^ Thus, UreNMs combine the key advantage of chemical enhancers (enhanced skin crossing) with that of NPs (efficient drug loading), overcoming the limitations of both approaches. Our findings pave the way to further studies in which enzymatic NMs could be explored as a vehicle for topical delivery of skin-relevant APIs, including small anticancer or anti-inflammatory molecules, as well as complex biologics such as peptides, proteins, or genetic material, for advanced therapeutic applications. Nonetheless, the loading and release of each API, as well as its transdermal delivery and biological activity when delivered by UreNMs, will need to be validated on a case-by-case basis.

With all this, we provided mechanistic insight into the ability of UreNMs to cross human skin, thereby supporting their potential application as transdermal drug delivery systems.^74,81,82^ The rearrangement of the lipid architecture of the SC induced by UreNMs (Figure 8E) is associated with an expansion of the lamellar spacing between corneocytes (Figure 8G), which facilitates the crossing of UreNMs across the skin (Figure 7).

## Conclusions

In summary, we have developed organic enzymatic NMs based on PLGA functionalized with urease for skin crossing. We thoroughly characterized UreNMs’ enzymatic activity and motility at the single and collective level. Additionally, we assessed their biocompatibility in a 3D model and their degradation in vitro. We reconstructed native human skin architecture in an in vitro model derived from donor-isolated cells. Then, we used the human model to confirm the crossing capability of UreNMs, observing a skin crossing efficiency of 15.7%. Using synchrotron radiation SAXS and electron microscopy, we revealed alterations in the lipidic organization of SC induced by the catalytic activity of UreNMs, supporting their potential for overcoming the skin barrier. Altogether, these results indicate that UreNMs combine the advantages of chemical penetration enhancers and NPs to cross human skin. By integrating local modulation of SC lipid structure through enzymatic reaction with increased active exploratory capability driven by active motion, UreNMs emerge as a promising platform for future transdermal drug delivery in humans.

## Methods and Experimental Details

### Materials

Starting materials were purchased from the following suppliers: Mowiol 4–88, ethyl acetate ACS ≥ 99.5%, fluorescein isothiocyanate (FITC) ≥ 90%, urease from *Canavalia ensiformis* (Jack bean) 50.000–100.000 units/g solid, ethylenediaminetetraacetic acid (EDTA) ACS 99.4–100.6%, 4-(hydroxymercuri)­benzoic acid sodium salt ≥ 95%, branched PEI 25.000 Mw, GA solution grade II 25% in H_2_O, Phenol Red ACS, formaldehyde solution (PFA) 36% in H_2_O, 9 mm diameter and 0.12 mm deep spacer Secure-Seal, Triton X-100, Urea ACS 99–100.5%, palmitic acid ≥ 99%, cholesterol ≥ 99%, methanol ≥ 99%, and Fluoroshield with DAPI, collagen IV, insulin, hydrocortisone, isoproterenol, KGF, l-carnitine, ascorbic acid, linoleic acid, arachidonic acid, vitamin E, vitamin C, 3-amino-9-ethylcarbazole, and 1,2-propylene oxide were purchased from Sigma-Aldrich. Poly­(lactic-*co*-glycolic acid) (PLGA) 50:50 24.000–38.000 Mw was purchased from BLDpharm. Cyanine5 NHS ester (Cy5) was purchased from Lumiprobe. Beta-mercaptoethanol 55 mM, Dulbecco’s modified Eagle medium (DMEM), Hams F12 medium, fetal bovine serum (FBS), penicillin–streptomycin 100x (10.000 U/mL), trypsin–EDTA 10x (0.5%), HBSS without Ca and Mg, and collagenase type 2 were purchased from Gibco (Thermo Fisher Scientific). 34 mm Petri dishes were purchased from Techno Plastic Products (TPP). SnakeSkin Dialysis Tubing 10.000 MWCO, low protein binding microcentrifuge tubes 2 mL, and Pierce BCA protein assay kit were purchased from Thermo Fisher Scientific. Millex polyethersulfone syringe filter 0.22 μm and Amicon ultra centrifugal filter 20 kDa MWCO were purchased from Millipore Merck. Rat tail collagen type-I 10 mg/mL was purchased from ibidi. Hoechst 33342 and ActinRed 555 were purchased from Invitrogen (Thermo Fisher Scientific). CellTiter-Glo 3D cell viability assay was purchased from Promega. C16 Ceramide (d18:1/16:0), cholesterol sulfate, filter support 10 mm, and PC membranes 0.1 μm were purchased from Avanti Research. Chloroform ≥ 99.8% was purchased from Avantor. Cytotoxicity Detection Kit (LDH) was purchased from Roche (Merck). Superfrost PLUS gold slides were purchased from Epredia. Dispase II was purchased from Roche Mannheim. Ultroser G was purchased from Sartorius. Transwells 0.4 μm, 24 mm were purchased from Corning. Acetic acid, formaldehyde, and BrightVision plus poly-HRP-antimouse/rabbit IgG were purchased from VWR. Fibrinogen was purchased from Diagnostica Stago. Antibody against Keratin 10 was purchased from Life Technologies. Antibody against Keratin 15 was purchased from Monosan. Antibody against Loricrin was purchased from Biolegend. Normal human dermal fibroblast (NHDF) cells were obtained from collaborators. The water used was type I quality (ultrapure water) obtained from a purification system (18.2 MΩ cm^–1^).

#### Synthesis of PLGA NPs

PLGA NPs were synthesized by an oil-in-water emulsion method, adapted from our previous work.^100^ An aqueous phase (10 mL) of 10 mg/mL Mowiol, poly­(vinyl alcohol) of 31.000 Mw, was dissolved by sonicating (20 min) and stirring (30 min). Anhydrous ethyl acetate (9 mL) was used as an organic phase to dissolve 30 mg of PLGA 50:50 24.000–38.000 Mw. The organic solution was added dropwise (5 mL/min) with a 21 G needle into the aqueous phase while stirring at 200 rpm. After the addition, a 3 mm tip sonicator (Branson) was used to homogenize the mixture for 1 min at 55% intensity. Finally, the NPs were solidified by evaporating the ethyl acetate in an open flask overnight (21 h) while continuously stirring at 200 rpm. For fluorescent labeling, 20 μL of Cyanine5 NHS ester (Cy5) (stock: 5 mg/mL in 25% DMSO and 75% Milli-Q water) was added immediately after the sonication step. For FITC labeling, 3 mg of FITC was added in the organic phase, and the same protocol was followed, except a 5 s tip sonication instead of 1 min.

#### Urease Chemical Inhibition

A triple chemical inhibition of urease from *C. ensiformis* (Jack bean) was performed with ethylenediaminetraacetic acid (EDTA), β-mercaptoethanol, and 4-(hydroxymercuri)­benzoic acid sodium salt (>95%). Urease was prepared at 5 mg/mL in PBS 1x, EDTA at 2 mM from a 0.5 M stock, β-mercaptoethanol at 4 mM from 55 mM stock, and 4-(Hydroxymercuri)­benzoic acid sodium salt at 600 μM from a 2 mM stock (dissolved in ethanol and sonicated 20 min). The solution was mixed for 24 h under rotational shaking, and the extra inhibitors were removed by dialysis in PBS 1x in a 10.000 MWCO SnakeSkin membrane (4 h + ON).

#### Functionalization with Urease/Inhibited Urease of the PLGA NP Surface

The surface of PLGA NPs was first modified by adding a positive layer of branched PEI. Low binding tubes were used for incubating 500 μL of PLGA NPs at 3 mg/mL with 50 μL of PEI at 1% v/v in Milli-Q-water (0.33% PEI/mg PLGA). PEI optimization was performed by incubating a range of PEI from 0 to 0.4%/mg PLGA. Incubation was kept under rotational shaking for 1 h 30 min at room temperature. Excess of PEI was removed by centrifuging the NPs at 3500*g* for 15 min at 4 °C. The pellet was resuspended in 480 μL of Milli-Q-water before adding 20 μL of GA 10% v/v (final concentration 0.4%). For the optimization, 4% GA was achieved by adding 20 μL of stock in 480 μL of NPs and 10% by adding 50 μL of stock in 450 μL of NPs. The reaction was kept for 1 h 30 min under rotatory shaking before centrifuging at 3500*g* for 15 min at 4 °C and removing the unbound GA. The resulting pellet was resuspended in 500 μL of urease or inhibited urease at 5 mg/mL in PBS 1x and kept overnight under rotatory shaking. Finally, inhNPs and UreNMs were cleaned twice by centrifuging at 3500 g for 15 min at 4 °C. Sterile inhNPs and UreNMs were produced by filtering all the individual components through a 0.22 μm PES membrane and working under laminar flow.

#### Morphology Characterization by Electron Microscopy

For the scanning electron microscopy (SEM), samples concentrated 10 times were dried onto a silicon wafer and covered with a thin layer of gold (10 nm) to enhance their visualization by increasing their conductivity (Leica EM ACE600 gold coater sputter, 30 mA current strength, and 2 min exposure). Images were obtained in a NOVA NanoSEM 230 (FEI) instrument with a voltage of 10 kV and analyzed using ImageJ V.1.54f. For transmission electron microscopy (TEM), NPs were adsorbed onto glow-discharged carbon-coated copper grids for 1 min and washed with Milli-Q water for another 1 min. Samples were negatively stained by floating the grid in a drop of 2% uranyl acetate for 1 min. The excess liquid was blotted manually from the edge of the grids. The samples were observed in a Tecnai Spirit transmission electron microscope (FEI, The Netherlands) equipped with a tungsten cathode. Images were acquired at 120 kV with a 1 kx1k Megaview III CCD camera. The negative staining technique and the observation of the samples were performed at the Cryo microscopy Unit from the CCiTUB. Images were analyzed by using ImageJ V.1.54f.

#### Hydrodynamic Diameter, Surface Charge, and Polydispersity Characterization

DLS (Malvern Zetasizer Advance Range Ultra) was used to measure hydrodynamic diameter and polydispersity of PLGA NPs, PLGA–PEI NPs, inhNPs, and UreNMs. Samples at 0.3 mg/mL were transferred to a 4 mL polystyrene cuvette (Sarstedt), and all measurements were conducted at a constant position of 4.64 mm and attenuation 6. The surface charge was quantified in the same Malvern Zetasizer Advance Range Ultra, but samples were transferred to a disposable capillary cell DTS1070 (Malvern) (measurements in Milli-Q water).

#### Quantification of the Surface Amount of Enzyme

The amount of urease present on the surface of NMs was indirectly quantified by measuring the unbound enzyme remaining in solution after functionalization. Supernatants of the two final washes of the functionalization process were collected and washed by centrifuging at 20.000*g* for 15 min at 4 °C to avoid the interference of remaining NPs. Pierce BCA protein assay kit and the manufacturer’s protocol were used for the analysis. Briefly, 25 μL of each sample (supernatants and initial urease solution) in triplicate was added into a 96-well plate and mixed with 200 μL of working solution of reagent A: reagent B (50:1). The plate was incubated for 30 min at 37 °C, and the absorbance was read at 562 nm (Synergy HTX Absorbance microplate reader). The concentration of urease in the NMs’ surface was calculated by interpolating the absorbance values into a calibration concentration curve (μg/mL) and subtracting the μg/mL of free urease to the μg/mL of initial urease.

#### Super-Resolution Microscopy Visualization of Surface Urease

UreNMs were functionalized, as previously described, but incorporating 20% of Cy5-Urease in the last functionalization step. Urease was labeled with melaimide-Cy5 by incubating free urease with the dye in a 1:10 ratio. The unbound Cy5 was removed with a PD-10 column Sephadex G-25 M and by dialysis (10000 MWCO). UreNMs for STORM visualization were sedimented for 15 min in a custom-made chamber before exchanging the solution for GLOX buffer. The GLOX buffer was composed of an oxygen-scavenging system (glucose oxidase at 0.5 mg/mL and catalase at 35 μg/mL), glucose (5% w/v), and β-mercaptoethylamine (100 mM). A Nikon N-STORM system with total internal reflection fluorescence was used for imaging the samples. The acquisition was performed for 10000 frames, and Cy5 was excited using a 647 nm laser.

#### Urease Enzymatic Activity Assay and Kinetic Characterization

Urease enzymatic activity tests were performed following our previously published protocol.^25^ Urea at different concentrations (0, 2.5, 10, 25, 50, 75, 100, 150, and 200 mM) were prepared in PBS pH 6.5 and with Phenol Red at 0.025 mM. For each sample (UreNMs and inhNPs at 2 mg/mL, free urease and free inhibited urease at 130 μg/mL), 2 μL was added to the bottom of a 96-well plate. Then, 200 μL of the desired urea concentration was mixed, and the absorbance was immediately measured at 560 nm under constant shaking at 37 °C for 1 h 30 min (Synergy HTX Absorbance microplate reader). The absorbance values obtained were transformed into phenol red concentration using the Beer–Lambert law (*A =* ε*cl*), where “*A”* is the absorbance measured, “ε*”* is the phenol red molar extinction coefficient (31620 M^–1^cm^–1^), “*c”* is the concentration (M), and “*l”* is the path length (0.5 cm). To further obtain the amount of ammonia generated, 1 mol of phenol red was considered equivalent to 2 mol of ammonium (stoichiometry 1:2). Values were normalized with time 0. The kinetic characterization of UreNMs was obtained by measuring the enzymatic activity at 0, 2.5, 10, 25, 50, 75, 100, 150, and 200 mM urea and fitting the values to a Michaelis–Menten curve (eq 1). The data were processed with GraphPad Prism v.10 to obtain the Michalis–Menten constant (*K*_m_), and the maximum velocity (*V*_max_).

1 $$V=V_{\max}\left[urea\right]/K_{m}+\left[urea\right]$$

#### Recording of Motion at the Single Particle Level and Analysis

inhNPs and UreNMs labeled with the fluorescent dye Cy5 were used for tracking their individual motion in 0 mM and 100 mM urea. Samples were diluted to ensure the visualization of single NPs and mixed with PBS or urea solution. A glass slide with a 9 mm diameter and 0.12 mm deep spacer was used to place 2 μL with the solution mix. The chamber was covered with a coverslip and visualized under a 100× magnification immersion objective in a fluorescent THUNDER microscope from LEICA equipped with a Leica K8 Scientific CMOS camera (635 nm laser excitation). Videos of 30 s at 9 FPS (minimum time interval) were recorded and processed with a homemade Python code, which extracted the individual trajectories and further calculated the mean square displacement (MSD, eq 2) and the diffusion coefficient (*D*_e_, eq 3).

2 $$MSD\left(\Delta t\right)={\left(x_{i}\left(t+\Delta t\right)-x_{i}\left(t\right)\right)}^{2}$$

3 $$MSD\left(\Delta t\right)=4D_{e}\Delta t$$

The above equations are valid for spherical NMs with nanometer-sized diameters, with low rotational diffusion over short time intervals. For all of the conditions, 30 particles were analyzed.

#### Recording of Motion at the Collective Level and Analysis

A fluorescent THUNDER microscope, exciting at 635 nm, from LEICA equipped with a Leica K8 Scientific CMOS camera and 1.25× objective was used for recording the collective displacement of UreNMs. 5 μL of Cy5-labeled inhNPs or UreNMs at 10 mg/mL was dispersed in a 34 mm Petri dish filled with 3 mL of PBS (0 mM urea) or 100 mM urea. Videos of 120 s at 8.5 FPS (minimum time interval) were recorded, and the covered area over time was extracted with a homemade Python code. The expanded area quantification considers the cumulative covered area in the previous frames, and it is normalized to the first frame. Side view videos were recorded with a homemade setup using a digital camera (Thorlabs, DCC1240M-GL/Thorlabs, CS165CU) equipped with a FUJINON HF35HA-1S lens. One-minute videos at 18 fps were recorded in polystyrene cuvettes (Sarstedt) with 2 mL of PBS or urea after adding 5 μL of UreNMs at 10 mg/mL at the bottom. The video analysis was performed in ImageJ V.1.54f, and the upward velocity was calculated as the slope (mm/s).

#### Degradation Studies of NMs

Three different batches of PLGA NPs and UreNMs were synthesized and prepared at 3 mg/mL for studying their degradation by OD, DLS, and SEM. For OD measurements, 1 mL of each replicate was transferred into a spectroscopy cuvette and kept at 37 °C under constant shaking for 15 weeks. NPs were dissolved in PBS, 100 mM urea, or in a solution containing the products of the reaction. The catalytic products were obtained after incubating 100 mM urea with free urease at 5 mg/mL (10:1) overnight and filtering the products from the remaining enzyme with an Amicon Ultra 30 k tube (Merck Millipore Ltd.). Every week, a NanoDrop spectrophotometer (BioDrop Duo) was used to read OD600 in triplicate. In a similar way, samples for DLS characterization were prepared by adding 1 mL of each replicate in a 4 mL polystyrene cuvette (Sarstedt), and every week, a Malvern Zetasizer Advance Range Ultra was used to measure (all measurements were conducted at a constant position of 4.64 mm and attenuation 6). Finally, SEM samples were prepared by concentrating each replicate 4 times and incubating them for 15 weeks at 37 °C under constant shaking. Each week, 5 μL of each replicate was dried onto a silicon wafer and sputtered with a 10 nm gold layer (Leica EM ACE600 gold coater sputter). A NOVA NanoSEM 230 (FEI) with a voltage of 10 kV was used to obtain images. After 13 weeks, samples were measured by SAXS at an NCD-SWEET beamline at the ALBA Synchrotron, Spain (Project ID 20250370417). The incident beam energy was set at 12.4 keV, with a distance between the sample and detector of 2.8 m (Pilatus 1 M detector, pixel size of 172 μm); under those conditions, the scattering vector *q* [*q* = 4π sin­(θ)/λ, where 2θ is the scattering angle and λ is the incoming wavelength] was from 0.05 to 1.5 nm^–1^. The measurements were performed at 22 °C. The liquid samples were placed in holders with Kapton windows. The SAXS patterns were fitted using a multilevel Guinier–Porod semiempirical model (10.1107/S0021889810015773). Each structural level was assumed to be spherical, with a Guinier slope fixed at 0. The Porod exponent was fixed at 4 for the small particles, consistent with smooth and compact interfaces. The larger structural contribution was approximated as the smallest particle size capable of representing the overall SAXS pattern. This approach was used to estimate the total scattering volume through the invariant (*Q*), as defined in eq 4.

4 $$Q=\int_{\cap }^{\infty }I\left(q\right)q^{2}dq$$

The Guinier–Porod function served as a parametrization tool to allow integration over the full *q*-range. In this way, the invariant (*Q*) represents a minimum estimate of the scattering volume and is used here as a comparative parameter between samples.

#### Generation of a 3D Collagen Matrix and Characterization by Refraction Microscopy and Immunostaining

NHDFs were kindly gifted by Giuseppe Battaglia’s group from IBEC and cultured in DMEM supplemented with 10% FBS and 1% penicillin–streptomycin. Cells were kept at 37 °C and 5% CO_2_ atmosphere and split when they reached 80% confluence. Rat tail collagen type I at 4 mg/mL was mixed with 6.5 × 10^4^ NHDF cells/mL and 2 μL of 1 M NaOH on ice. 300 μL of the mix was added to 48-well plates and polymerized by incubating for 30 min at 37 °C. Then, 500 μL of complete DMEM media was added on top, and cells were grown for 3 days at 37 °C and 5% CO_2_ atmosphere. To visualize UreNMs in the collagen matrix, 50 μg/mL of FITC-labeled NMs was added with 100 mM urea and incubated for 1 h before washing and adding fresh media. After 24 h of culture, samples were fixed with 3.4% paraformaldehyde (PFA) for 30 min at room temperature at 4 °C. PFA was removed, and samples were cleaned twice in PBS 1x. A Zeiss LSM 800 confocal microscope equipped with a ×40 immersion objective was used to illuminate at 647 nm and detect the refracted light by the unlabeled collagen fibers. In addition, a laser at 488 nm was used to visualize FITC-UreNMs at the same time. Samples were imaged in 3D with a Z-stack and further analyzed and projected (average intensity) with ImageJ V.1.54f. Fixed collagen models with and without FITC-UreNMs were permeabilized with Triton X-100 0.5% for 45 min and cleaned twice with PBS 1x. Then, samples were stained with Hoechst 33342 at 5 μg/mL for nuclei visualization and with ActinRed-555 at 3 drops/mL for F-actin labeling. Incubation was kept at room temperature and in the dark for 2 h and cleaned three times with PBS 1x. Samples were imaged in 3D with the same Zeiss LSM 800 confocal microscope and further processed with ImageJ V.1.54f.

#### Biocompatibility Studies in a 3D Collagen Matrix

Models of NHDFs in a 3D collagen matrix were prepared as previously described and incubated with sterile UreNMs and inhNPs at a final concentration of 0, 5, 30, 50, and 150 μg/mL in both the presence and absence of urea (0 mM and 100 mM, respectively). After 1 h of incubation at 37 °C and 5% CO_2_, NMs were removed and fresh media added for another 24 h. Then, media were removed and replaced with 400 μL of CellTiter-Glo 3D cell viability assay prepared in a 1:1 dilution with DMEM supplemented media. Samples were shaken vigorously at room temperature for 40 min and then left static for 10 min for signal stabilization. Finally, triplicates of 100 μL were transferred to a 96-well plate, and luminescence was read with a FLUOstar Omega plate reader. Viability percentages were normalized with the 0 μg/mL UreNMs/inhNPs in 0 mM urea controls (100% viability), and the statistical analysis was performed between each group.

#### Cell Culture of Fibroblasts and KCs from Human Donors

Human skin from healthy donors was collected as surgical waste after abdominal dermolipectomy, classified as “non-Medical Research Involving Human Subjects Act” (non-WMO). The tissue was used in an anonymous fashion, and all donors gave informed consent, as described in the “Code of Conduct for Health Research” as formulated by COREON (Committee on Regulation of Health Research; https://www.coreon.org). This study protocol was reviewed and approved by the METC of Amsterdam UMC (Amsterdam, The Netherlands), approval number 2012.248. KCs were isolated from human skin as follows: adipose tissue was removed before separating the dermis and the epidermis. The skin was cut into small pieces (5 mm × 5 mm) and incubated in Dispase II (1.9 U/ml) overnight at 4 °C. The next day, skin pieces were placed at 37 °C for 15 min, and epidermal sheets were carefully removed from the dermis using forceps, washed 3x with PBS, and incubated with 0.05% trypsin for 15 min at 37 °C. After trypsin neutralization, cells were vigorously shaken 3 times and subsequently filtered over 100 and 40 μm cell strainers. Cells were centrifuged, counted, resuspended to 4 × 10^5^ cells/ml, and plated on a collagen IV-coated Petri dish and cultured for 4 h at 37 °C, 7.5% CO_2_, in KC Medium I (375 mL DMEM with 125 mL Hams F12 with the following additives: 1% UltroserG, 0.1 μM insulin, 1 μM hydrocortisone, 1 μM isoproterenol, and 1% pen/strep). After 4 h, medium was aspirated and replaced with KC1 medium + KC growth factor (KGF, 2 ng/mL), and cells were grown to confluency with medium exchange at day 3 or 4. Cells were used at passage 1 or 2.

Dermal fibroblasts were isolated from human skin as follows: after removal of the epidermal sheets, the dermis pieces were incubated for 2 h at 37 °C with collagen/Dispase solution (2.5 mL of Dispase, 7.5 mL of HBSS without Ca and Mg, and 75 mg of collagenase type 2) with regular mixing. After 2 h, the enzymatic activity was then neutralized by adding fibroblast medium (DMEM with 1% UltoserG, 1% pen/strep) to the tissue-enzyme solution. Cell suspension was centrifuged (300*g*, 5 min, 4 °C), resuspended in fibroblast medium, and subsequently filtered over 100 and 40 μm cell strainers. Cells were centrifuged (300*g*, 5 min, 4 °C), seeded at 1–3 × 10^6^ cells/dish in 10 mL fibroblast medium, and incubated at 37 °C and 5% CO_2_. The next day, cells were washed once with PBS and supplemented with fresh fibroblast medium, and cells were grown to confluency with medium exchange at day 3 or 4. Cells were used at passage 3.

#### Reconstructed Human Skin (RhS)

RhS was constructed in transwells (8 μm, Brand GmbH). For the dermis layer, 6 mg/mL collagen (isolated from rat tails and dissolved in 0.1% acetic acid) was mixed with 2 mg/mL fibrinogen in a ratio of 1:1, and 6.5 × 10^4^ dermal fibroblasts were added to 1 mL of hydrogel. For fibrin formation and polymerization, 0.5 U/ml thrombin was added, and the hydrogel was cast (500 μL/transwell). After 48 h, 1.5 × 10^5^ KCs were seeded on top, and culture medium was replaced with KC1 medium supplemented with 2 ng/mL KGF. Skin constructs were cultured and submerged for 3 days, at 37 °C and 7.5% CO_2_ from now on and subsequently cultured at the air–liquid interface in KC medium II [KCII: DMEM/Ham’s F12 3:1, supplemented with 1% pen/strep, 0.2% UltroserG, 1 μM hydrocortisone, 1 μM isoproterenol hydrochloride, 0.1 μM insulin, 10 μM l-carnitine, 10 mM l-serine (Sigma-Aldrich), 50 μg/mL ascorbic acid, and 2 ng/mL KGF]. Cultures were kept at the air–liquid interface for 14 days and incubated in KCII without hydrocortisone 24 h before exposure to NPs. To increase differentiation of SC, part of the samples were incubated in KCII supplemented with a lipid mixture (palmitic acid (25 μM), linoleic acid (15 μM), arachidonic acid (7 μM), 24 μmol/L bovine serum albumin (Sigma-Aldrich), and 1 μM Vit-E, 0.4 mM Vit-C).

#### Histology

For histology, tissue samples were fixed overnight in 4% formaldehyde before preparation for histological analysis. Formalin-fixed paraffin-embedded 5 μm-thick tissue sections were stained with hematoxylin and eosin (H&E) to check for overall histology and morphology. In addition, sections were stained with antibodies against Keratin 10 (K10, clone DE-K10), Keratin 15 (K15, clone EPR1614Y), and Loricrin (polyclonal) as follows. Sections for K15 and Loricrin were immersed in 10 mM citrate buffer (pH 6) for 15 min at 100 °C, followed by slow cooling down to RT. For K10 sections, samples were incubated with 50 μL of pepsin (0.1–0.3%) for 10 min at 37 °C. All samples were washed with PBS and incubated with the primary antibody for 1 h at RT, followed by incubation with BrightVision plus poly-HRP-antimouse/rabbit IgG and 3-amino-9-ethylcarbazole substrate and by a counterstain with hematoxylin. Stained tissue sections were imaged using the VS200 slide scanner (Olympus, Tokyo, Japan).

#### Addition of NMs in RhS

Human skin models from 3 different donors were prepared as previously explained. For each biological replicate, between 2 and 3 models (technical replicates) were generated. Solutions of sterile Cy5-UreNMs/Cy5-inhNPs at 0/300 μg/mL and 0/100 mM urea were mixed just before addition into the models. A drop of 25 μL was carefully added in the middle of the skin avoiding reaching the edge of model. Then, samples were incubated for 24 h at 37 °C in a 5% CO_2_ atmosphere. Before fixation, the remaining drop was removed by pipetting, and the constructs were delicately removed from the transwell and membrane. Supernatants in the basolateral compartment were kept for further analysis. Models were cut by half; one piece was transferred to a paraffin cassette in formalin for staining, and the other was put into a 24-well plate with 2 mL of 4% PFA EM grade.

#### Effect of NMs on RhS

After UreNM/inhNP incubation in the skin models as described in “Addition of NMs in RhS”, the tissues in paraffin were sectioned and stained with H&E as reported in “Histology”. For the viability measurement, supernatants after incubation were collected and LDH was measured as a cytotoxicity indicator. The test was performed following the commercial protocol (Cytotoxicity Detection Kit (LDH), Roche), where the samples were incubated with a catalyst + dye solution for 20 min before reading the absorbance at 490 nm. Values were extrapolated in a standard curve to determine the LDH concentration released by the skin models after exposure.

#### Human Skin Crossing Studies

Samples were obtained as explained in the section “Addition of NMs in RhS” of Methods. After 1 h of fixation in PFA 4%, samples were cleaned in PBS and transferred to a well with 15% sucrose for 2 h at 4 °C. Subsequently, 15% sucrose was replaced for a 30% solution for an overnight incubation at 4 °C. After samples were well embedded into sucrose to avoid cryopreservation artifacts, models were moved to a cryomold and filled with OCT medium before freezing into liquid nitrogen. Samples were kept at −80 °C degrees prior to cryosectioning. Longitudinal 15 μm slices of all tissues were performed and deposited onto Superfrost adhesion PLUS gold slides (Epredia). Cuts were conducted from the dermis to the epidermis to minimize the perturbation of SC architecture. Slides were filled with 4–5 tissue slices until almost all tissue was represented (5–6 slides). When the slices were dried and well attached to the slides, Fluoroshield with DAPI (4′,6-diamidino-2-phenylindole) was added and homogeneously dispersed by covering the samples with a coverslip. Slides were incubated overnight at room temperature to allow the mounting media to dry. Finally, a fluorescence scanner (Olympus SlideView VS200) was used to image all the obtained slides. Complete images of the longitudinal skin models’ cuts were used for the analysis. QuPath v5.0.1 software was used to quantify the mean Cy5 fluorescence detected in three different regions of interest (ROI): SC, viable epidermis, and dermis. The different ROIs were differentiated by their unique cellular densities detected with the DAPI staining. The mean values were transformed to absolute fluorescence intensity and used to calculate the Cy5 percentage of fluorescence in each ROI. Skin crossing (SC overcoming) was calculated as the fluorescence detected in the viable epidermis and in the dermis.

#### SC Lipid Model

The protocol followed for generating models of the SC lipidic composition was adapted from the work of Roy et al.^85^ Stock solutions of Ceramide 3C16 (d18:1/16:0) (N-palmitoyl-D-*erythro*-sphingosine), palmitic acid, cholesterol, and cholesterol 3-sulfate were prepared in chloroform–methanol 2:1 in volume. A mol-percent mixture of 40% ceramide 3, 25% palmitic acid, 25% cholesterol, and 10% cholesterol sulfate was transferred to a round-bottom flask, and the solvent was removed under high vacuum (15 mbar) in a rotatory evaporator (Hei-VAP Expert/ultimate) at room temperature. To ensure a completely dried thin layer, the flask was connected to a vacuum pump and kept for 1 h at 0.01 mbar of pressure. Multilamellar vesicles were formed by hydrating the lipidic film with PBS 1x at 80 °C for 2 h under constant mixing. Uniform unilamellar membranes were obtained after extruding the vesicles through a 100 nm polycarbonate membrane filter at 80 °C 21 times (extruding heating block from Avanti Polar Lipids Inc.). Characterization of the resulting vesicles was performed by DLS of a 1/10 dilution.

#### Effect of NMs on the SC Lipid Model

SC lipid models were prepared, as previously mentioned, and incubated with inhNPs and UreNMs in the absence (0 mM) and presence (100 mM) of urea, as well as with the products of the reaction (PR) alone. PR were obtained by incubating 100 mM urea with urease at 5 mg/mL (10:1) overnight and filtering the products from the remaining enzyme with an Amicon Ultra 30 k tube (Merck Millipore Ltd.). Sample measurements were conducted by SAXS at the NCD-SWEET beamline at the ALBA Synchrotron, Spain (Project ID 2024098683). The incident beam energy was set at 15 keV, with a distance between the sample and detector of 6.6 m (Pilatus 1 M detector, pixel size of 172 μm); under those conditions, the scattering vector *q* (*q* = 4π sin­(θ)/λ, where 2θ is the scattering angle and λ is the incoming wavelength) was from 0.05 to 1.5 nm^–1^. The measurements were performed at 22 °C. The liquid samples were placed in holders with Kapton windows. The bilayer form factor was modeled as the summation of thin planar layers with defined electron density profiles to capture the scattering contrast across the bilayer cross-section. Assuming a symmetric bilayer, the Fourier transform of the electron density can be expressed as represented in equation 5..

5 $$P\left(q\right)=\frac{1}{q^{2}}{\left[\int_{l}^{-l}\rho \left(R’\right)\cos\left(qR’\right)dR^{\prime }\right]}^{2}$$

where *l* is the integration limit, ρ­(*R*′) = 0 if |*R*′| > *l*. In this particular case, the electron density function was approximated by the contribution of three Gaussians,^86^ two for the polar head of the amphiphile molecules and another for the hydrocarbon core (hc).

6 $$\rho \left(R^{\prime }\right)=Sc_{ph}Gauss\left(R^{\prime }-R_{ph},\sigma_{ph}\right)+Sc_{hc}Gauss\left(0,\sigma_{hc}\right)+Sc_{ph}Gauss\left(R^{\prime }+R_{ph},\sigma_{ph}\right)$$

In order to model the data, a symmetric Gaussian-based electron density profile was used with two Gaussians representing the head groups with identical amplitudes (*Sc*_ph_) and one Gaussian representing the hydrocarbon core (*Sc*_hc_ = −1).^87^ The distance between the centers of the two polar head Gaussians that model the headgroup shell was set to the sum of half-width half-maximum of polar and hydrophobic groups [ $R_{ph}=\sqrt{2\ln\left(2\right)}\left(\sigma_{ph}+\sigma_{hc}\right)$ ], whereas the sigma value was kept as a fit parameter to diminish artifacts from nonlinear parameter correlation.

The final scattering intensity was described as in eq 7

7 $$I\left(q\right)={scale}_{1}\cdot P\left(q\right)+{scale}_{2}/q^{p}+background$$

where scale_1_, scale_2_, and background are scalars, and the parameter *p* is the Porod exponent that corresponds to the surface scattering from the spherical liposome structure, where 3 < *p* ≤ 4.^96^

#### SC Electron Microscopy Imaging

The samples were fixed in 3% GA and 3% PFA. Before embedding, regions of interest were cut out and postfixed with 1% osmium tetroxide (OsO4) for 1 h. Subsequently, samples were dehydrated in an alcohol series from 70% until 100%, and the last steps in 1,2-propylene oxide, then in a 1:1 ratio of 1,2-propylene oxide/Epon (LX-112 resin, Ladd Research), and finally embedded in full Epon and polymerized at 65 °C. Ultrathin (60 nm) Epon sections were generated using a diamond (Diatome) knife on a Leica Ultracut UC7 microtome, collected on slots 2 × 0.5 mm copper grids (FF205-Cu; Aurion), and counterstained with uranyl acetate and lead citrate. All samples were examined and photographed using a Jeol JEM 1400 flash transmission electron microscope with a Xarosa camera and Radius software (EMSIS). The preparation and the observation of the samples were performed at the Cellular Imaging core facility of the Amsterdam UMC (location AMC). Images were analyzed using ImageJ V.1.54f.

## Supplementary Material









## Notes

Nanomotors characterization by DLS and SEM, chemical inhibition of urease and its enzymatic activity characterization, mean square displacement and diffusion coefficient enhancement of urease-powered nanomotors, side view characterization of swarming behavior, nanomotors degradation studies in aqueous solution, characterization of interdonor variability in human skin models, viability of human skin models after incubation with urease-nanomotors, human skin crossing studies, human skin crossing studies in lipid-supplemented models, and SAXS analysis of stratum corneum model after incubation with products of the reaction (PDF)
Collective behavior of active urease-nanomotors in the presence of urea 100 mM (MP4)
Collective behavior of active urease-nanomotors in the absence of urea (0 mM) (MP4)
Collective behavior of passive inhibited urease-nanoparticles in the presence of urea 100 mM (MP4)

## References

[1] Baker P., Huang C., Radi R., Moll S. B., Jules E., Arbiser J. L. (2023). Skin Barrier Function: The Interplay of Physical, Chemical,
and Immunologic Properties. Cells.

[2] Bos J. D., Meinardi M. M. H. M. (2000). The 500 Da Rule for the Skin Penetration of Chemical
Compounds and Drugs. Exp. Dermatol..

[3] Lane M. E. (2013). Skin Penetration
Enhancers. Int. J. Pharm..

[4] Yu Y. Q., Yang X., Wu X. F., Fan Y. B. (2021). Enhancing
Permeation
of Drug Molecules Across the Skin via Delivery in Nanocarriers: Novel
Strategies for Effective Transdermal Applications. Front. Bioeng. Biotechnol..

[5] Bolzinger M. A., Briançon S., Pelletier J., Chevalier Y. (2012). Penetration
of Drugs through Skin, a Complex Rate-Controlling Membrane. Curr. Opin. Colloid Interface Sci..

[6] Dehkharghani S., Bible J., Chen J. G., Feldman S. R., Fleischer A. B. (2003). The Economic
Burden of Skin Disease in the United States. J. Am. Acad. Dermatol..

[7] Hay R. J., Johns N. E., Williams H. C., Bolliger I. W., Dellavalle R. P., Margolis D. J., Marks R., Naldi L., Weinstock M. A., Wulf S. K., Michaud C., J.l. Murray C., Naghavi M. (2014). The Global Burden of Skin Disease in 2010: An Analysis
of the Prevalence and Impact of Skin Conditions. J. Invest. Dermatol..

[8] Hadgraft J., Lane M. E. (2016). Advanced Topical Formulations (ATF). Int. J. Pharm..

[9] Ramadon D., McCrudden M. T. C., Courtenay A. J., Donnelly R. F. (2022). Enhancement Strategies
for Transdermal Drug Delivery Systems: Current Trends and Applications. Drug Delivery Transl. Res..

[10] Ghaferi M., Alavi S. E., Phan K., Maibach H., Mohammed Y. (2024). Transdermal
Drug Delivery Systems (TDDS): Recent Advances and Failure Modes. Mol. Pharm..

[11] Akram M. W., Jamshaid H., Rehman F. U., Zaeem M., Khan J., Zeb A. (2022). Transfersomes: A Revolutionary
Nanosystem for Efficient Transdermal
Drug Delivery. AAPS PharmSciTech.

[12] Moghadam S. H., Saliaj E., Wettig S. D., Dong C., Ivanova M. V., Huzil J. T., Foldvari M. (2013). Effect of Chemical
Permeation Enhancers
on Stratum Corneum Barrier Lipid Organizational Structure and Interferon
Alpha Permeability. Mol. Pharm..

[13] Marwah H., Garg T., Goyal A. K., Rath G. (2016). Permeation Enhancer
Strategies in Transdermal Drug Delivery. Drug
Deliv..

[14] Tiwari N., Osorio-Blanco E. R., Sonzogni A., Esporrín-Ubieto D., Wang H., Calderón M. (2022). Nanocarriers for Skin Applications:
Where Do We Stand?. Angew. Chem. Int. Ed..

[15] Mitchell M. J., Billingsley M. M., Haley R. M., Wechsler M. E., Peppas N. A., Langer R. (2021). Engineering
Precision Nanoparticles for Drug Delivery. Nat.
Rev. Drug Discov..

[16] Watkinson A. C., Bunge A. L., Hadgraft J., Lane M. E. (2013). Nanoparticles Do
Not Penetrate Human Skin - A Theoretical Perspective. Pharm. Res..

[17] Hansen S., Lehr C. M. (2012). Nanoparticles for
Transcutaneous Vaccination. Microb. Biotechnol..

[18] Antony A., Raju G., Job A., Joshi M., Shankarappa S. (2024). Penetration
of Topically Applied Polymeric Nanoparticles across the Epidermis
of Thick Skin from Rat. Biomed. Phys. Eng. Express.

[19] Matharoo N., Mohd H., Michniak-Kohn B. (2024). Transferosomes
as a Transdermal Drug
Delivery System: Dermal Kinetics and Recent Developments. Wiley Interdiscip Rev Nanomed Nanobiotechnol.

[20] Ruiz-González N., Esporrín-Ubieto D., Kim I. D., Wang J., Sánchez S. (2025). Micro- and Nanomotors: Engineered Tools for Targeted
and Efficient Biomedicine. ACS Nano.

[21] Chen S., Fan D. E., Fischer P., Ghosh A., Göpfrich K., Golestanian R., Hess H., Ma X., Nelson B. J., Patiño
Padial T., Tang J., Villa K., Wang W., Zhang L., Sen A., Sánchez S. (2025). A Roadmap
for Next-Generation Nanomotors. Nat. Nanotechnol..

[22] Ruiz-González N., Sánchez-DeAlcázar D., Esporrín-Ubieto D., Di Carlo V., Sánchez S. (2025). Hyaluronic
Acid-Based Nanomotors:
Crossing Mucosal Barriers to Tackle Antimicrobial Resistance. ACS Appl. Mater. Interfaces.

[23] Ruiz-González N., Esporrín-Ubieto D., Hortelao A. C., Fraire J. C., Bakenecker A. C., Guri-Canals M., Cugat R., Carrillo J. M., Garcia-Batlletbó M., Laiz P., Patiño T., Sánchez S. (2024). Swarms of
Enzyme-Powered Nanomotors Enhance the Diffusion
of Macromolecules in Viscous Media. Small.

[24] Serra-Casablancas M., Di Carlo V., Esporrín-Ubieto D., Prado-Morales C., Bakenecker A. C., Sánchez S. (2024). Catalase-Powered
Nanobots for Overcoming
the Mucus Barrier. ACS Nano.

[25] Esporrín-Ubieto D., Ruiz-González N., Di Carlo V., Sánchez-deAlcázar D., Lezcano F., Pushkareva Fazullina A., Sánchez S. (2026). Smart Nanogels
as Enzyme-Driven Nanomotors for Navigating Viscous Physiological Barriers. Adv. Funct. Mater..

[26] Ramos
Docampo M. A., Wang N., Pendlmayr S., Städler B. (2022). Self-Propelled Collagenase-Powered Nano/Micromotors. ACS Appl. Nano Mater..

[27] Patiño T., Feiner-Gracia N., Arqué X., Miguel-López A., Jannasch A., Stumpp T., Schäffer E., Albertazzi L., Sánchez S. (2018). Influence of Enzyme Quantity and
Distribution on the Self-Propulsion of Non-Janus Urease-Powered Micromotors. J. Am. Chem. Soc..

[28] Hortelão A. C., Patiño T., Perez-Jiménez A., Blanco À., Sánchez S. (2018). Enzyme-Powered Nanobots Enhance Anticancer
Drug Delivery. Adv. Funct. Mater..

[29] Patino T., Arqué X., Mestre R., Palacios L., Sánchez S. (2018). Fundamental
Aspects of Enzyme-Powered Micro- and Nanoswimmers. Acc. Chem. Res..

[30] Arqué X., Andrés X., Mestre R., Ciraulo B., Ortega Arroyo J., Quidant R., Patiño T., Sánchez S. (2020). Ionic Species
Affect the Self-Propulsion of Urease-Powered Micromotors. Research.

[31] Arqué X., Patiño T., Sánchez S. (2022). Enzyme-Powered Micro- and Nano-Motors:
Key Parameters for an Application-Oriented Design. Chem. Sci..

[32] Fichna K., Crespo-Cuadrado M., Konuparamban A., Di Carlo V., Esporrín-Ubieto D., Macías-Tarrío I., Jutglar Soler O., Chen S., Gómez-Martínez M., Bakenecker A. C., Vilaseca A., Llop J., Sánchez S. (2026). Nanomotor-Assisted
Intravesical Chemotherapy for Bladder Tumor Reduction and Suppression
of Early Tumor Regrowth. Nano Lett..

[33] Ji X., Yang H., Liu W., Ma Y., Wu J., Zong X., Yuan P., Chen X., Yang C., Li X., Lin H., Xue W., Dai J. (2021). Multifunctional Parachute-like
Nanomotors for Enhanced Skin Penetration and Synergistic Antifungal
Therapy. ACS Nano.

[34] Liu L., Li S., Yang K., Chen Z., Li Q., Zheng L., Wu Z., Zhang X., Su L., Wu Y., Song J. (2023). Drug-Free
Antimicrobial Nanomotor for Precise Treatment of Multidrug-Resistant
Bacterial Infections. Nano Lett..

[35] Li Y., Qian L., Liu F., Xu S., Zhou L., Wei C., Zhang Y., Zhai Y., Gu Y., Li S. (2025). Light-Controlled
Small Extracellular Vesicle-Based Spherical Nucleic Acid Nanomotor
for Enhanced Transdermal Delivery against Skin Aging. Nano Lett..

[36] Agarwal Y., Beatty C., Ho S., Thurlow L., Das A., Kelly S., Castronova I., Salunke R., Biradar S., Yeshi T., Richardson A., Bility M. (2020). Development of Humanized
Mouse and Rat Models with Full-Thickness Human Skin and Autologous
Immune Cells. Sci. Rep..

[37] Gallagher S., Kruger U., Josyula K., Rahul, Gong A., Song A., Sweet R., Makled B., Parsey C., Norfleet J., De S. (2022). Thermally Damaged Porcine Skin Is Not a Surrogate Mechanical Model
of Human Skin. Sci. Rep..

[38] Sintov A. C. (2017). Cumulative
Evidence of the Low Reliability of Frozen/Thawed Pig Skin as a Model
for in Vitro Percutaneous Permeation Testing. Eur. J. Pharm. Sci..

[39] Gerber P. A., Buhren B. A., Schrumpf H., Homey B., Zlotnik A., Hevezi P. (2014). The Top Skin-Associated
Genes: A Comparative Analysis
of Human and Mouse Skin Transcriptomes. Biol.
Chem..

[40] Rimal R., Muduli S., Desai P., Marquez A. B., Möller M., Platzman I., Spatz J., Singh S. (2024). Vascularized 3D Human
Skin Models in the Forefront of Dermatological Research. Adv. Healthc. Mater..

[41] Hofmann E., Schwarz A., Fink J., Kamolz L. P., Kotzbeck P. (2023). Modelling
the Complexity of Human Skin In Vitro. Biomedicines.

[42] Quílez C., Bebiano L. B., Jones E., Maver U., Meesters L., Parzymies P., Petiot E., Rikken G., Risueño I., Zaidi H., Zidarič T., Bekeschus S., van den Bogaard E. H., Caley M., Colley H., López N. G., Letsiou S., Marquette C., Maver T., Pereira R. F., Tobin D. J., Velasco D. (2024). Targeting the Complexity of In Vitro
Skin Models: A Review of Cutting-Edge Developments. J. Invest. Dermatol..

[43] Ponec M., Gibbs S., Pilgram G., Boelsma E., Koerten H., Bouwstra J., Mommaas M., Ponec M. (2001). Barrier Function
in
Reconstructed Epidermis and Its Resemblance to Native Human Skin. Skin Pharmacol. Appl. Skin Physiol..

[44] Wang Y., Qin B., Xia G., Choi S. H. (2021). FDA’s Poly (Lactic-Co-Glycolic
Acid) Research Program and Regulatory Outcomes. AAPS J..

[45] Alsaab H. O., Alharbi F. D., Alhibs A. S., Alanazi N. B., Alshehri B. Y., Saleh M. A., Alshehri F. S., Algarni M. A., Almugaiteeb T., Uddin M. N., Alzhrani R. M. (2022). PLGA-Based
Nanomedicine: History
of Advancement and Development in Clinical Applications of Multiple
Diseases. Pharmaceutics.

[100] Fraire J. C., Prado-Morales C., Aldaz Sagredo A., Caelles A. G., Lezcano F., Peetroons X., Bakenecker A. C., Di Carlo V., Sánchez S. (2024). Swarms of
Enzymatic Nanobots for Efficient Gene Delivery. ACS Appl. Mater. Interfaces.

[46] Todaria M., Awasthi R. (2025). PLGA Nanoparticles
as Promising Drug Delivery Carrier:
The Future of Skin Cancer Treatment. J.Umm Al-Qura
Univ. Appll. Sci..

[47] Das S., Khuda-Bukhsh A. R. (2016). PLGA-Loaded
Nanomedicines in Melanoma Treatment: Future
Prospect for Efficient Drug Delivery. Indian
J. Med. Res..

[48] Luengo J., Schneider M., Schneider A. M., Lehr C. M., Schaefer U. F. (2021). Human Skin
Permeation Enhancement Using Plga Nanoparticles Is Mediated by Local
Ph Changes. Pharmaceutics.

[49] Takeuchi I., Kagawa A., Makino K. (2020). Skin Permeability and
Transdermal
Delivery Route of 30-Nm Cyclosporin A-Loaded Nanoparticles Using PLGA-PEG-PLGA
Triblock Copolymer. Colloids Surf. A Physicochem.
Eng. Asp..

[50] Arqué X., Torres M. D. T., Patiño T., Boaro A., Sánchez S., de la Fuente-Nunez C. (2022). Autonomous
Treatment of Bacterial Infections *in Vivo* Using Antimicrobial
Micro- and Nanomotors. ACS Nano.

[51] Piquero-Casals J., Morgado-Carrasco D., Granger C., Trullàs C., Jesús-Silva A., Krutmann J. (2021). Urea in Dermatology: A Review of
Its Emollient, Moisturizing, Keratolytic, Skin Barrier Enhancing and
Antimicrobial Properties. Dermatol. Ther..

[52] Liu L., Yu W., Seitsonen J., Xu W., Lehto V. P. (2022). Correct Identification
of the Core-Shell Structure of Cell Membrane-Coated Polymeric Nanoparticles. Chem.—Eur. J..

[53] Howse J. R., Jones R. A. L., Ryan A. J., Gough T., Vafabakhsh R., Golestanian R. (2007). Self-Motile Colloidal Particles: From Directed Propulsion
to Random Walk. Phys. Rev. Lett..

[54] Patiño T., Llacer-Wintle J., Pujals S., Albertazzi L., Sánchez S. (2024). Unveiling
Protein Corona Formation around Self-Propelled
Enzyme Nanomotors by Nanoscopy. Nanoscale.

[55] Golestanian R., Liverpool T. B., Ajdari A. (2005). Propulsion of a Molecular Machine
by Asymmetric Distribution of Reaction Products. Phys. Rev. Lett..

[56] De
Corato M., Arqué X., Patinõ T., Arroyo M., Sánchez S., Pagonabarraga I. (2020). Self-Propulsion
of Active Colloids via Ion Release: Theory and Experiments. Phys. Rev. Lett..

[57] Chen S., Peetroons X., Bakenecker A. C., Lezcano F., Aranson I. S., Sánchez S. (2024). Collective Buoyancy-Driven Dynamics in Swarming Enzymatic
Nanomotors. Nat. Commun..

[58] Patiño
Padial T., Chen S., Hortelão A. C., Sen A., Sánchez S. (2025). Swarming Intelligence in Self-Propelled
Micromotors and Nanomotors. Nat. Rev. Mater..

[59] Hortelao A. C., Simó C., Guix M., Guallar-Garrido S., Julián E., Vilela D., Rejc L., Ramos-Cabrer P., Cossío U., Gómez-Vallejo V., Patiño T., Llop J., Sánchez S. (2021). Swarming Behavior and in Vivo Monitoring
of Enzymatic Nanomotors within the Bladder. Sci. Robot..

[60] Chen S., Prado-Morales C., Sánchez-DeAlcázar D., Sánchez S. (2024). Enzymatic Micro/Nanomotors in Biomedicine: From Single
Motors to Swarms. J. Mater. Chem. B.

[61] Muthu M. S. (2009). Nanoparticles
Based on PLGA and Its Co-Polymer: An Overview. Asian J. Pharm..

[62] Amini-Fazl M. S. (2022). Biodegradation
Study of PLGA as an Injectable in Situ Depot-Forming Implant for Controlled
Release of Paclitaxel. J. Polym. Sci..

[63] Li T., Senesi A. J., Lee B. (2016). Small Angle
X-Ray Scattering for
Nanoparticle Research. Chem. Rev..

[64] Hussein A. S., Abdullah N., Ahmadun F. R. (2013). In Vitro
Degradation of Poly (D,
L-Lactide-Co-Glycolide) Nanoparticles Loaded with Linamarin. IET Nanobiotechnol..

[65] Zhang H., Yang Z., Wu D., Hao B., Liu Y., Wang X., Pu W., Yi Y., Shang R., Wang S. (2023). The Effect of Polymer Blends on the In Vitro Release/Degradation
and Pharmacokinetics of Moxidectin-Loaded PLGA Microspheres. Int. J. Mol. Sci..

[66] Machatschek R., Schulz B., Lendlein A. (2018). The Influence of PH on the Molecular
Degradation Mechanism of PLGA. MRS Adv..

[67] Sun R., Chen Y., Pei Y., Wang W., Zhu Z., Zheng Z., Yang L., Sun L. (2024). The Drug Release of
PLGA-Based Nanoparticles and Their Application in Treatment of Gastrointestinal
Cancers. Heliyon.

[68] Michielon E., Boninsegna M., Waaijman T., Fassini D., Spiekstra S. W., Cramer J., Gaudriault P., Kodolányi J., de Gruijl T. D., Homs-Corbera A., Gibbs S. (2024). Environmentally Controlled
Microfluidic System Enabling Immune Cell Flow and Activation in an
Endothelialised Skin-On-Chip. Adv. Healthc.
Mater..

[69] Bik L., van Doorn M. B. A., Biskup E., Ortner V. K., Haedersdal M., Olesen U. H. (2021). Electronic Pneumatic Injection-Assisted Dermal Drug
Delivery Visualized by Ex Vivo Confocal Microscopy. Lasers Surg. Med..

[70] Xia D., Jin R., Byagathvalli G., Yu H., Ye L., Lu C.-Y., Saad Bhamla M., Yang C., Prausnitz M. R. (2021). An Ultra-Low-Cost
Electroporator with Microneedle Electrodes (EPatch) for SARS-CoV-2
Vaccination. Proc. Natl. Acad. Sci. U. S. A..

[71] Bakhrushina E. O., Shumkova M. M., Avdonina Y. V., Ananian A. A., Babazadeh M., Pouya G., Grikh V. V., Zubareva I. M., Kosenkova S. I., Krasnyuk I. I., Krasnyuk I. I. (2025). Transdermal
Drug Delivery Systems:
Methods for Enhancing Skin Permeability and Their Evaluation. Pharmaceutics.

[72] Vahav I., Thon M., van den
Broek L. J., Spiekstra S. W., Atac B., Lindner G., Schimek K., Marx U., Gibbs S. (2022). Proof-of-Concept Organ-on-Chip
Study: Topical Cinnamaldehyde Exposure
of Reconstructed Human Skin with Integrated Neopapillae Cultured under
Dynamic Flow. Pharmaceutics.

[73] Dirschka T. (2020). Mode of Action
of Urea. Int. J. Clin. Pract..

[74] Mueller J., Oliveira J. S. L., Barker R., Trapp M., Schroeter A., Brezesinski G., Neubert R. H. H. (2016). The Effect of Urea and Taurine as
Hydrophilic Penetration Enhancers on Stratum Corneum Lipid Models. Biochim. Biophys. Acta Biomembr..

[75] Kumar P. K., Kumar R. S. (2021). Review on Transferosomes
and Transferosomal Gels. J. Pharm. Res. Int..

[76] Bouwstra J. A., Nădăban A., Bras W., McCabe C., Bunge A., Gooris G. S. (2023). The Skin
Barrier: An Extraordinary
Interface with an Exceptional Lipid Organization. Prog. Lipid Res.

[77] Elias P. M. (2005). Stratum
Corneum Defensive Functions: An Integrated View. J. Invest. Dermatol..

[78] Moore D. J., Rerek M. E., Mendelsohn R. (1997). FTIR Spectroscopy
Studies of the
Conformational Order and Phase Behavior of Ceramides. J. Phys. Chem. B.

[79] Boncheva M., Damien F., Normand V. (2008). Molecular Organization of the Lipid
Matrix in Intact Stratum Corneum Using ATR-FTIR Spectroscopy. Biochim. Biophys. Acta Biomembr..

[80] Moore D. J., Rerek M. E. (2000). Insights into the
Molecular Organization of Lipids
in the Skin Barrier from Infrared Spectroscopy Studies of Stratum
Corneum Lipid Models. Acta Derm Venereol Suppl
(Stockh).

[81] Obata Y., Utsumi S., Watanabe H., Suda M., Tokudome Y., Otsuka M., Takayama K. (2010). Infrared Spectroscopic
Study of Lipid
Interaction in Stratum Corneum Treated with Transdermal Absorption
Enhancers. Int. J. Pharm..

[82] Novotný M., Klimentová J., Janůšová B., Palát K., Hrabálek A., Vávrová K. (2011). Ammonium Carbamates
as Highly Active Transdermal Permeation Enhancers with a Dual Mechanism
of Action. J. Controlled Release.

[83] Madsen H. B., Ifversen P., Madsen F., Brodin B., Hausser I., Nielsen H. M. (2009). In Vitro Cutaneous
Application of ISCOMs on Human Skin
Enhances Delivery of Hydrophobic Model Compounds through the Stratum
Corneum. AAPS J..

[84] ertz W., W P., Abraham W., Landmann L., Downing D. T. (1986). Preparation of Liposomes
from Stratum Corneum Lipids. J Invest Dermatol.

[85] Roy S., Ho J. C. S., Teo D. L. C., Gupta S., Nallani M. (2023). Biomimetic
Stratum Corneum Liposome Models: Lamellar Organization and Permeability
Studies. Membranes (Basel).

[86] Tomsen-Melero J., Passemard S., García-Aranda N., Díaz-Riascos Z. V., González-Rioja R., Nedergaard Pedersen J., Lyngsø J., Merlo-Mas J., Cristóbal-Lecina E., Corchero J. L., Pulido D., Cámara-Sánchez P., Portnaya I., Ionita I., Schwartz S., Veciana J., Sala S., Royo M., Córdoba A., Danino D., Pedersen J. S., González-Mira E., Abasolo I., Ventosa N. (2021). Impact of Chemical Composition on
the Nanostructure and Biological Activity of Α-galactosidase-loaded
Nanovesicles for Fabry Disease Treatment. ACS
Appl. Mater. Interfaces.

[87] Alcaina-Hernando M., Malvacio I., Ferraboschi I., Huck-Iriart C., Bianchera A., Sala S., Pedersen J. S., Ferrer-Tasies L., Pescina S., Sissa C., Ventosa N., Córdoba A. (2024). A New Plant-Based
Drug Delivery Platform Based on Alkyl Polyglucosides and β-Sitosterol
Nanovesicles for Topical Delivery. Appl. Mater.
Today.

[88] Groen D., Gooris G. S., Bouwstra J. A. (2009). New Insights
into the Stratum Corneum
Lipid Organization by X-Ray Diffraction Analysis. Biophys. J..

[89] Hatta I. (2022). Stratum Corneum
Structure and Function Studied by X-Ray Diffraction. Dermato.

[90] Brooks S. G., Mahmoud R. H., Lin R. R., Fluhr J. W., Yosipovitch G. (2025). The Skin Acid
Mantle: An Update on Skin PH. J. Invest. Dermatol..

[91] Ananthapadmanabhan K.
P., Lips A., Vincent C., Meyer F., Caso S., Johnson A., Subramanyan K., Vethamuthu M., Rattinger G., Moore D. J. (2003). Ph-Induced Alterations in Stratum
Corneum Properties. Int. J. Cosmet. Sci..

[92] Lu G., Moore D. J. (2012). Study of Surfactant-Skin
Interactions by Skin Impedance
Measurements. Int. J. Cosmet. Sci..

[93] Choi E. H., Kang H. (2024). Importance of Stratum
Corneum Acidification to Restore Skin Barrier
Function in Eczematous Diseases. Ann. Dermatol..

[94] Paliwal S., Menon G. K., Mitragotri S. (2006). Low-Frequency
Sonophoresis: Ultrastructural
Basis for Stratum Corneum Permeability Assessed Using Quantum Dots. J. Invest. Dermatol..

[95] Choi H., Jeong S. H., Simó C., Bakenecker A., Liop J., Lee H. S., Kim T. Y., Kwak C., Koh G. Y., Sánchez S., Hahn S. K. (2024). Urease-Powered Nanomotor
Containing STING Agonist for Bladder Cancer Immunotherapy. Nat. Commun..

[96] Klein, L. ; Aparicio, M. ; Jitianu, A. Handbook of Sol-Gel Science and Technology; Springer: Cham, 2018.

