# Supplementary material for: Enhancing Human Skin Penetration with Biodegradable Enzymatic Nanomotors
Source: ACS Nano. 2026 Aug 4;20(32):23013–33. doi: 10.1021/acsnano.6c11619 (PMC13492477; doi:10.1021/acsnano.6c11619)
Supplement: Supplementary file 1 [file nn6c11619_si_001.pdf]

Enhancing Human Skin Penetration with Biodegradable Enzymatic Nanomotors

Carles Prado-Morales[1,2], Taco Waaijman[3], Inés Macías-Tarrío[1,2,4], Cristián Huck-Iriart[5], Tiziana Russo[1,6], Joël Gálvez-Savoca[1], Cesar Rodriguez-Emmenegger[1,7,8], Jasper J. Koning[3,9], Samuel Sánchez[1,8]**

[1] *Institute for Bioengineering of Catalonia (IBEC), Barcelona Institute of Science and Technology (BIST), 08028 Barcelona, Spain*

[2] *Universitat de Barcelona, Doctorate in Biotechnology, Facultat de Farmàcia i Ciències de l'Alimentació, 08028 Barcelona, Spain*

[3] *Department of Molecular Cell Biology and Immunology, Amsterdam UMC, Location VUMC, 1081 HV Amsterdam, The Netherlands*

[4] *Universitat de Barcelona, Departament de Bioquímica i Fisologia, Facultat de Farmàcia i Ciències de l'Alimentació, 08028 Barcelona, Spain*

[5] *ALBA Synchrotron Light Source, Cerdanyola del Vallès, 08290 Barcelona, Spain*

[6] *Universitat de Barcelona, Facultat de Biologia, 08028 Barcelona, Spain*

[7] *DWI – Leibniz Institute for Interactive Materials, 52074 Aachen, Germany*

[8] *Institució Catalana de Recerca i Estudis Avançats (ICREA), 08010 Barcelona, Spain*

[9] *Amsterdam institute for Immunology and Infectious diseases, Inflammatory diseases, 1081 HV Amsterdam, The Netherlands*

Key Words: Nanomotors, Nanobots, Nanomedicine, Biological Barrier, Skin, Transdermal Delivery, Human Skin Model

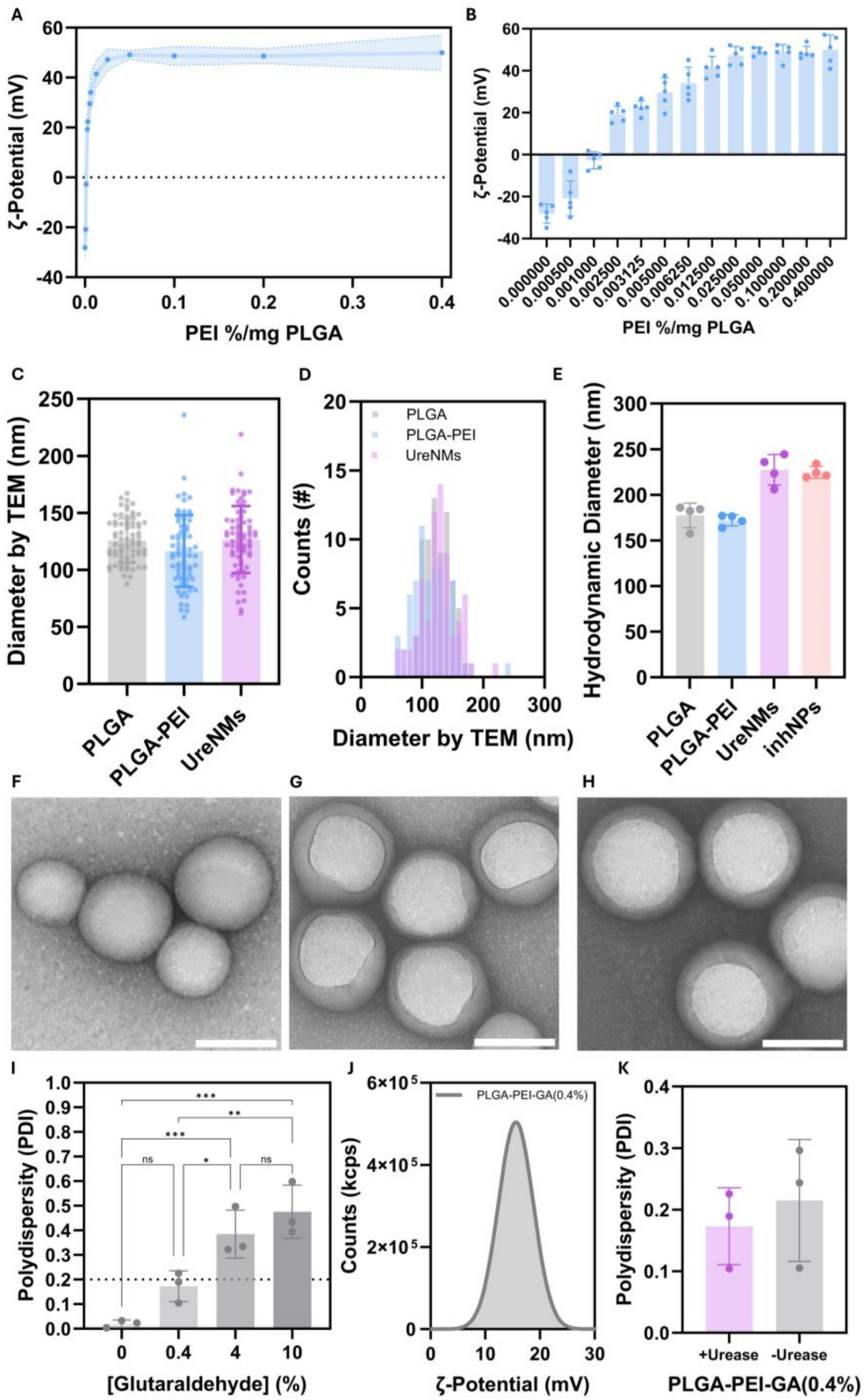

Figure S1. Nanomotors characterization. *(**A**) (**B**) Surface charge of PLGA-PEI NPs incubated with different amounts of polymer. (**C**) (**D**) Diameter of PLGA, PLGA-PEI and PLGA-PEI-Urease (UreNMs) were analyzed with TEM (n = 150). (**E**) Size was further measured by DLS to characterize the mean hydrodynamic diameter (n = 4). Representative TEM images were selected for PLGA (**F**), PLGA-PEI (**G**) and UreNMs (**H**) (scale bar = 50 nm). (**I**) UreNMs were functionalized with glutaraldehyde at different concentrations and its polydispersity measured by DLS (n = 3). Surface charge of PLGA-PEI incubated with GA at 0.4% (**J**) and its PDI in absence and presence of urease (**K**) was measured (n = 3). Results are shown as mean ± SD. * p < 0.05, ** p < 0.01, *** p < 0.001.*

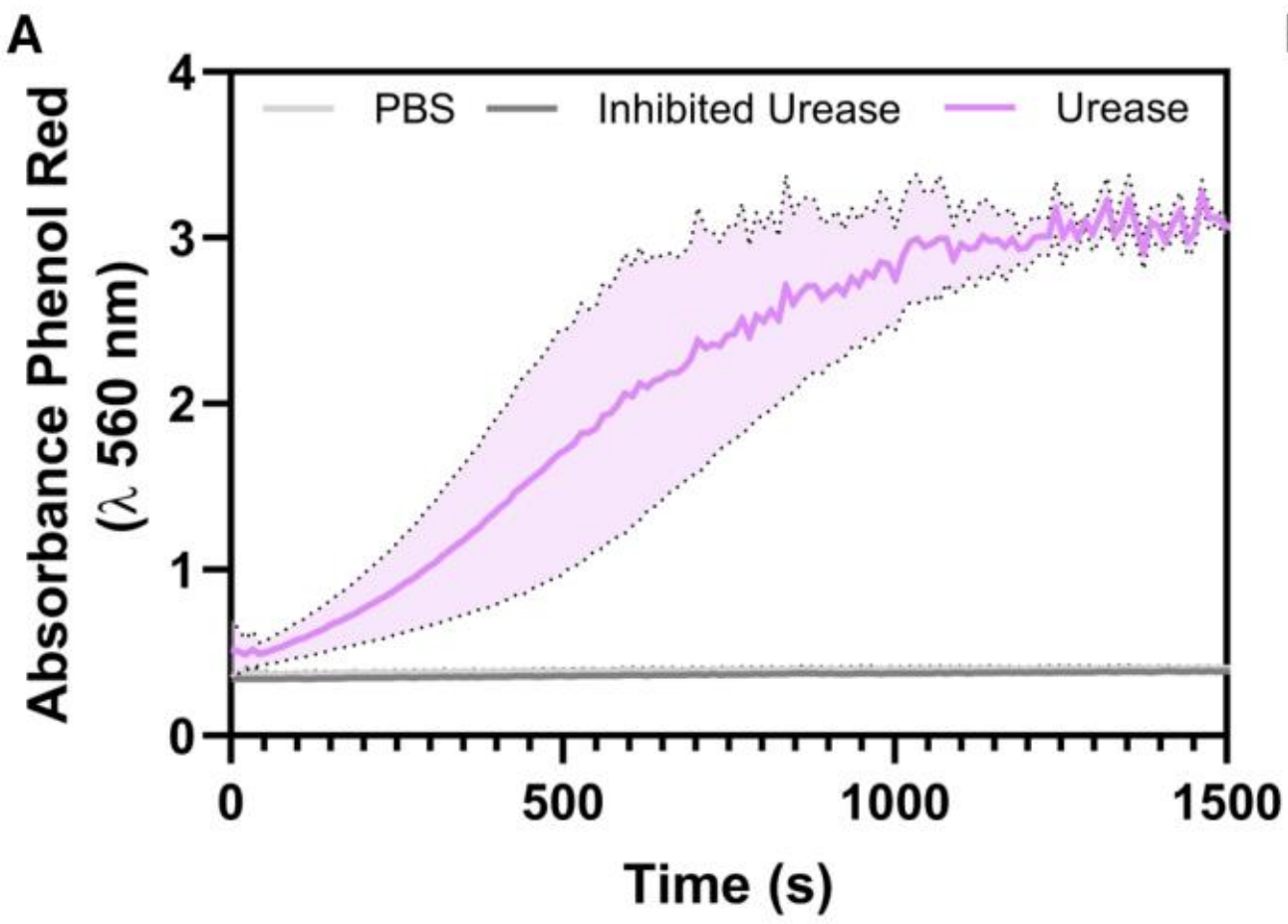

Figure S2. Chemical inhibition of urease. *(**A**) Enzymatic activity of free urease and free inhibited urease was checked by measuring the change in phenol red absorbance as consequence of pH increase. (**B**) Values at 1000 seconds were exported and compared between conditions. (n = 3 ± SD) **** p < 0.0001.*

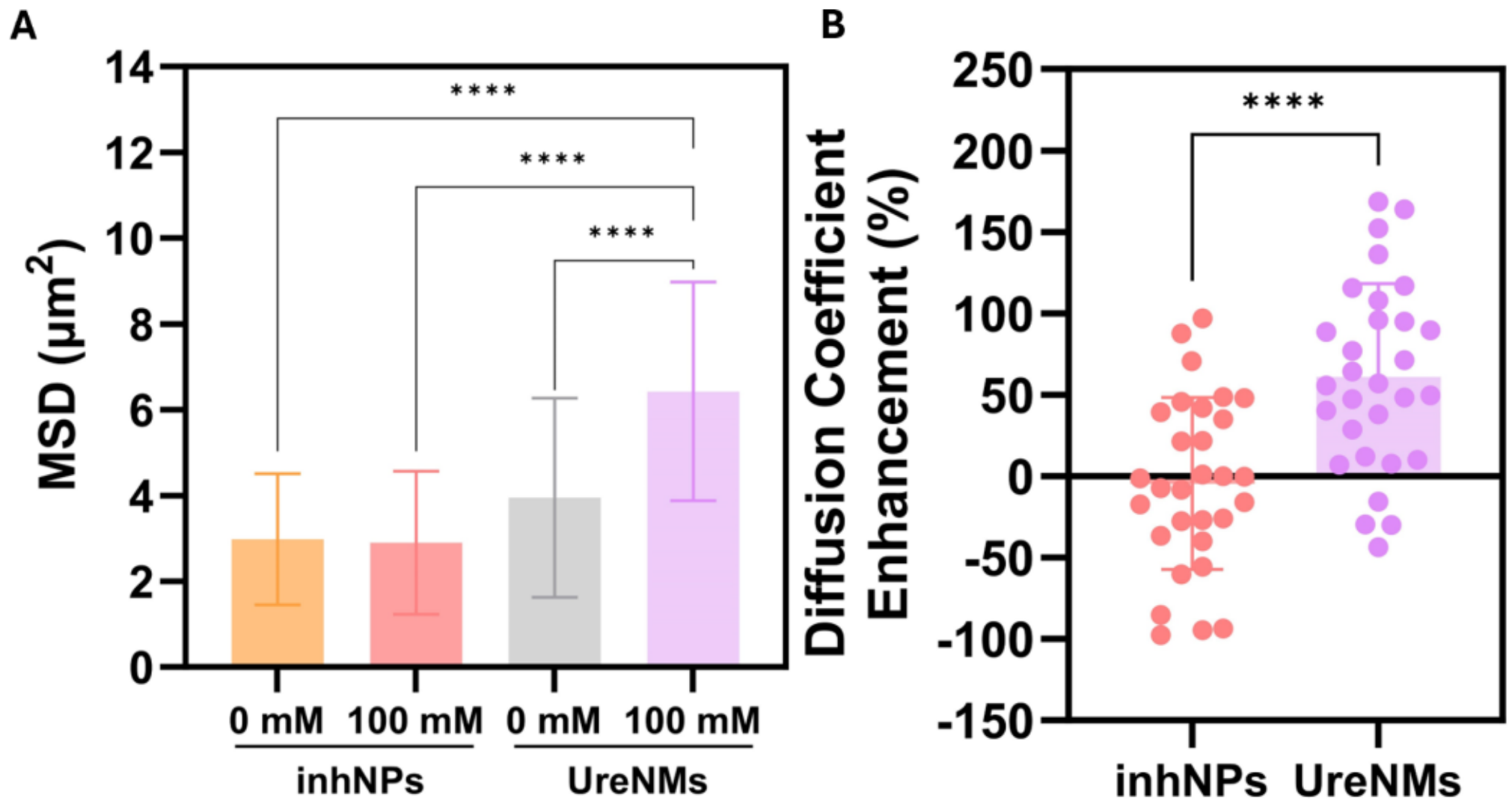

Figure S3. Nanomotors single particle motion. (***A***) *Individual inhNPs and UreNMs were incubated with 0 mM or 100 mM urea and their trajectories were recorded. From them, the MSD at second 2 was plotted and compared (n = 30) **** p < 0.0001.* (***B***) *The enhancement of diffusion coefficient of UreNMs and inhNPs was calculated comparing the values obtained in 100 mM urea with the average of the respective control at 0 mM urea (n = 30). Results are shown as mean ± SD. **** p < 0.0001.*

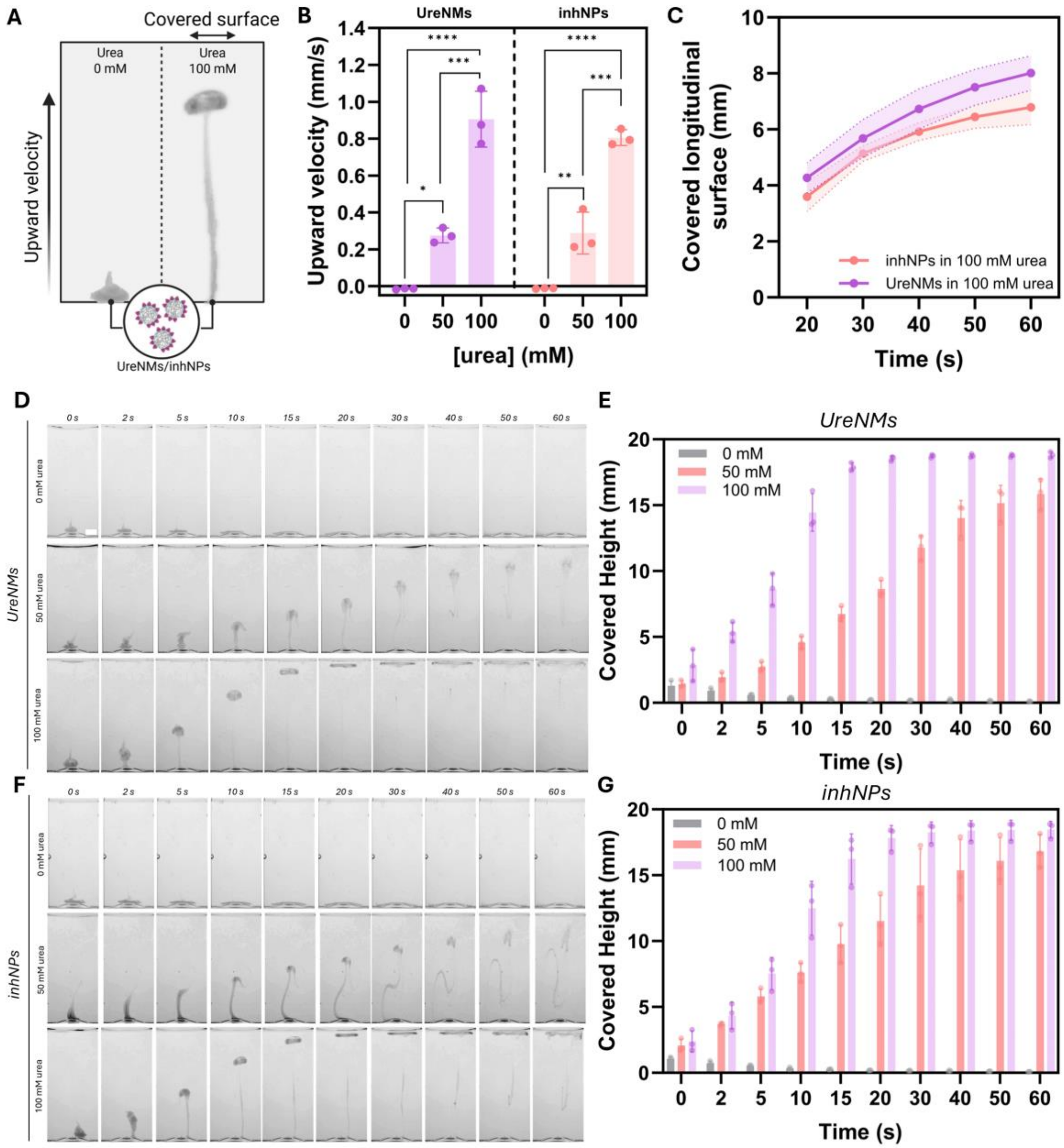

Figure S4. Side view characterization of swarming behavior. *(**A**) Scheme of the experiment where the upward velocity of the swarm was recorded in absence and presence of urea 100 mM. (**B**) The upward velocity of active UreNMs and passive inhNPs was measured at 0, 50 and 100 mM urea (n = 3 ± SD). (**C**) The longitudinal covered are by both types of NPs was measured from time 20 seconds to 1 minute. The side view recorded videos of UreNMs (**D**) (**E**) and inhNPs (**F**) (**G**) in 0, 50 and 100 mM urea were represented with a representative screenshot (scale bar = 2mm) and used to measure the height of the front of the swarm at different time points in 0, 50 and 100 mM urea (n = 3). Results are shown as mean ± SD. * $p < 0.05$, ** $p < 0.01$, *** $p < 0.001$, **** $p < 0.0001$. Created in BioRender. Prado Morales, C. (2026) https://BioRender.com/ue90du3.*

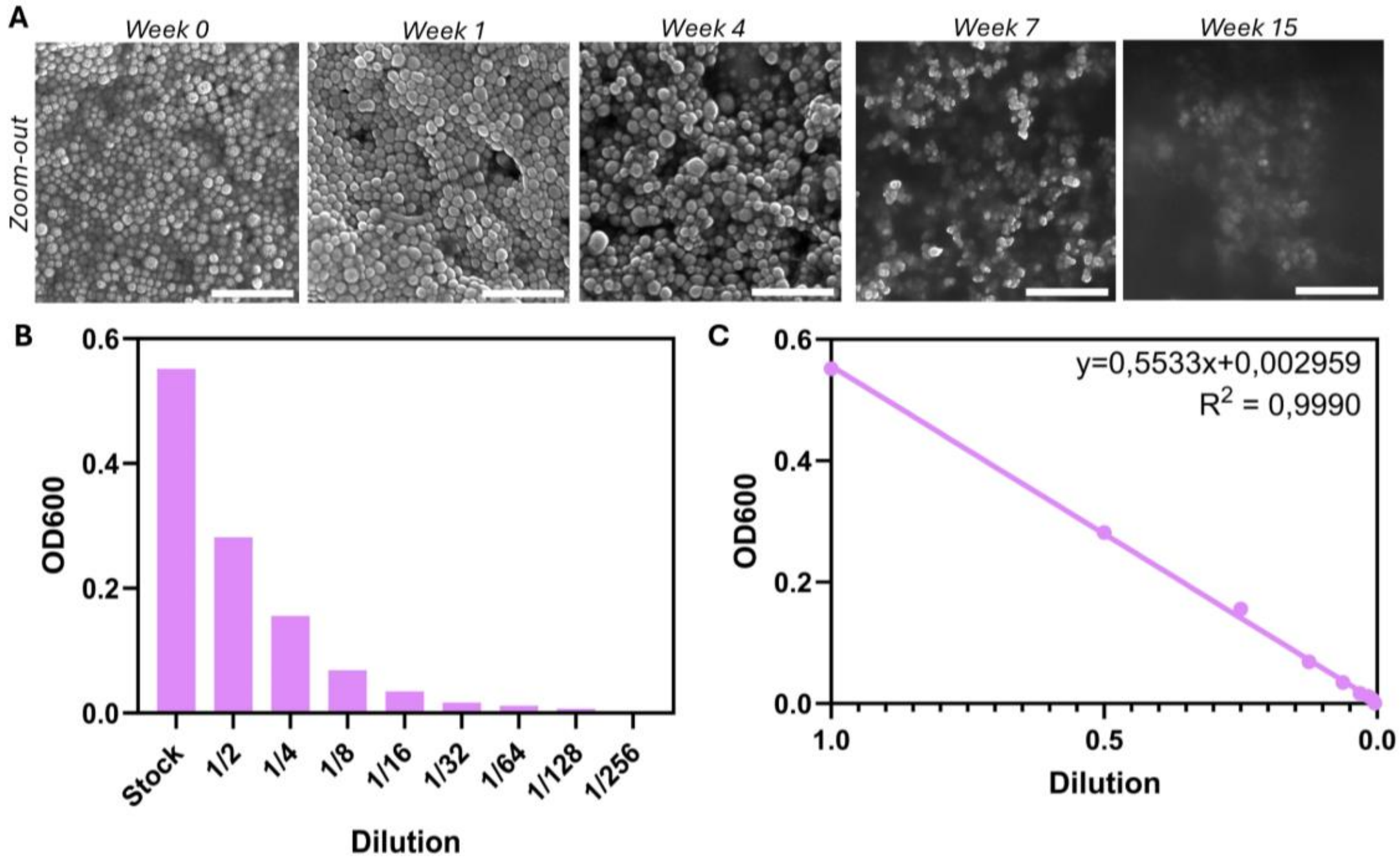

Figure S5. Nanomotors degradation. *(**A**) UreNMs in water were kept at 37 ° C and their morphology was checked by SEM every week (zoom-out scale bar = 1000 nm) (n = 3). (**B**) A stock solution of PLGA NPs was diluted by ½ until 1/256 and we measured their optical density at 600 nm. (**C**) Same values were adjusted to a linear regression.*

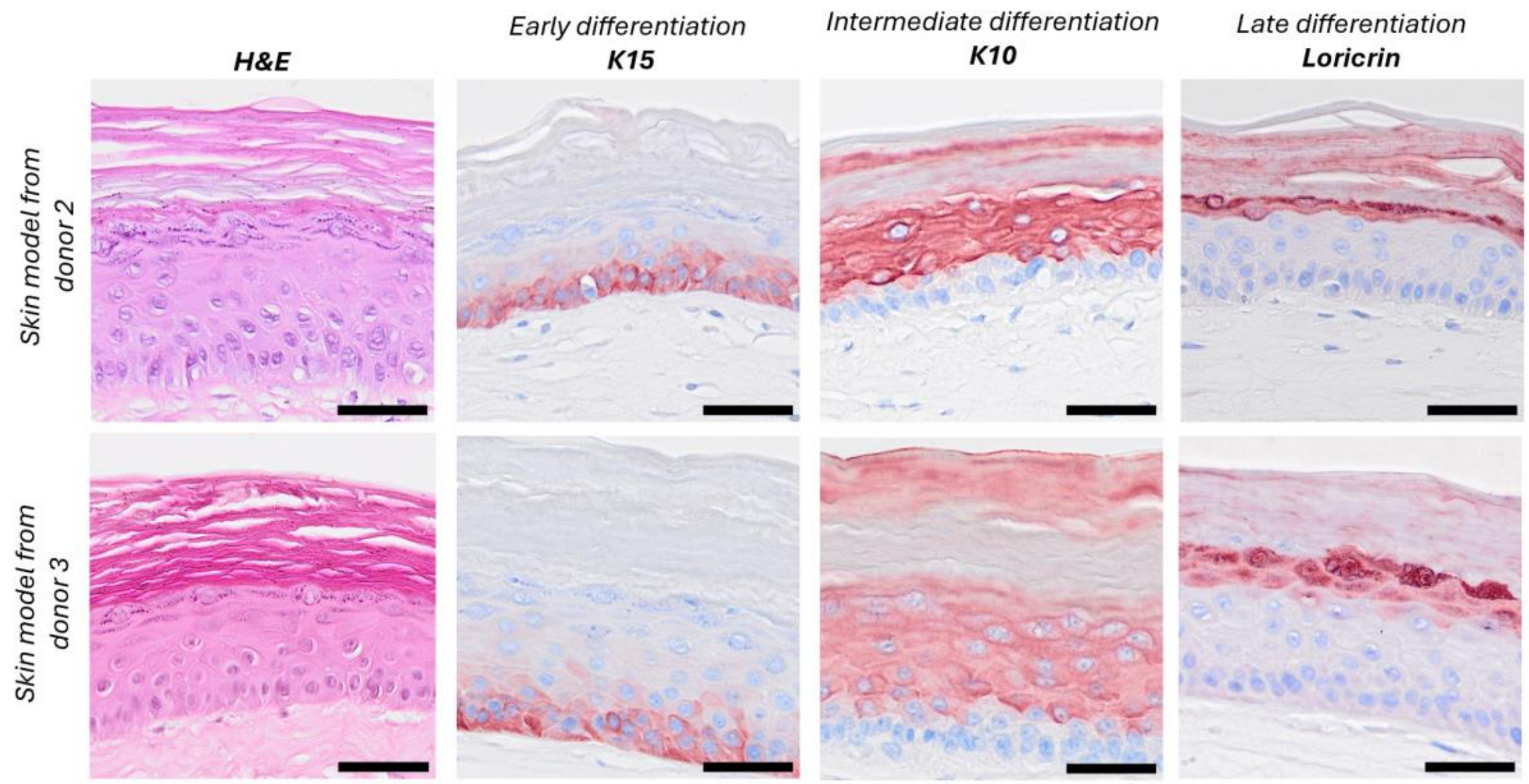

Figure S6. Characterization of inter-donor variability of skin models. *Skin architecture of skin models generated with cells isolated from donor 2 and donor 3 was characterize by H&E and immunohistochemistry against keratin 15 (K15, as basal layer marker), keratin 10 (K10, as differentiation suprabasal epidermis marker) and Loricrin (as terminal differentiation marker) (scale bar = 50 µm).*

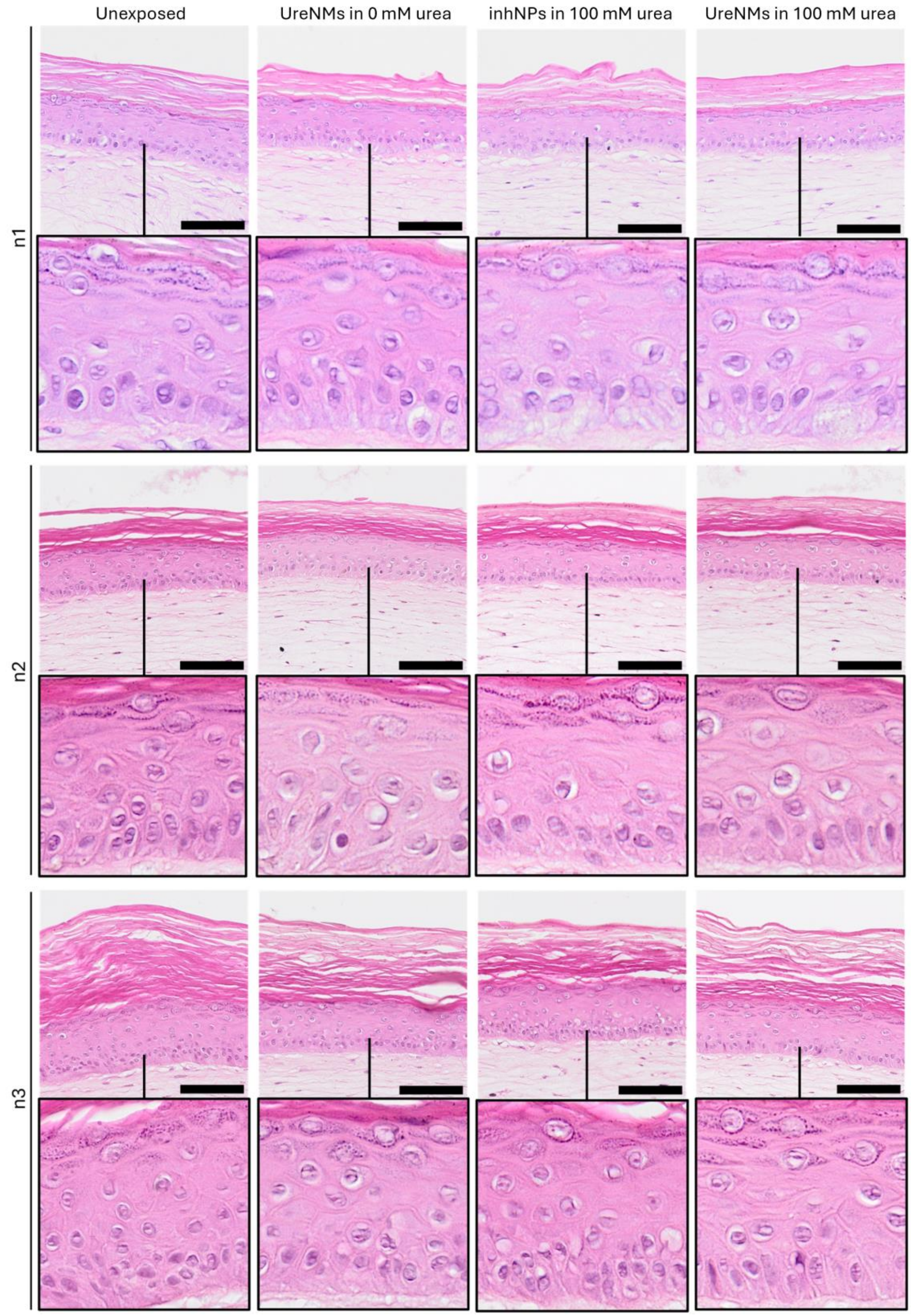

Figure S7. Viability of skin models after incubation with UreNMs. *UreNMs and inhNPs at 300 µg/mL with 0 mM and 100 mM urea were added into the top of the skin models and incubated for 24 h. Then, affections in the epidermal skin architecture were checked by H&E staining for each biological replicate (scale bar = 100 µm).*

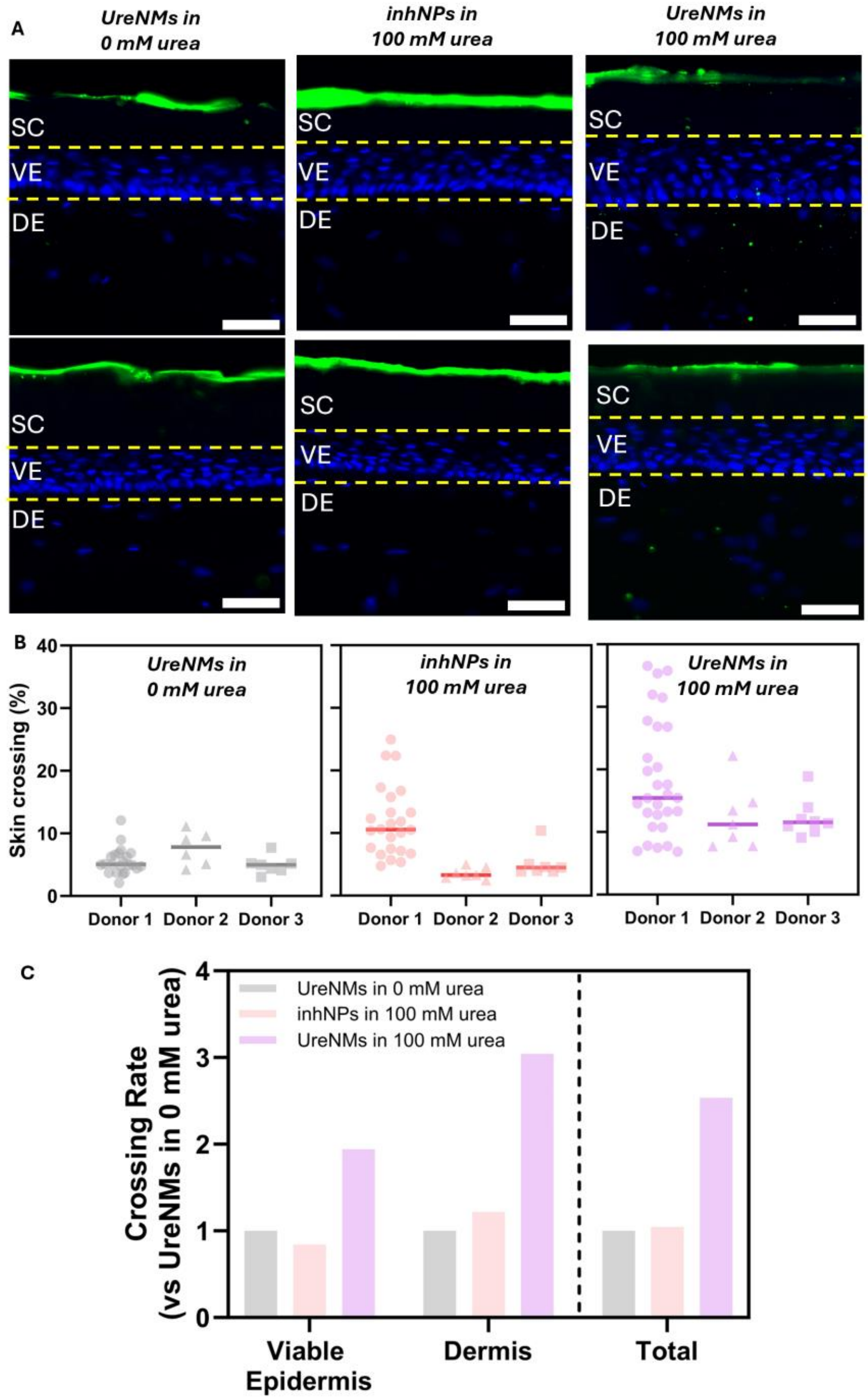

Figure S8. Human skin crossing studies. (***A***) *In vitro human skin models were developed and a drop of Cy5-UreNMs or Cy5-inhNPs (green) was topically applied in absence (0 mM) or presence (100 mM) of urea and incubated 24h. Then, cells nuclei were stained with DAPI (blue) and imaged by fluorescence. Representative images were selected for each condition and yellow dotted lines used for differentiating the 3 ROIs: Stratum Corneum (SC), viable epidermis (VE) and dermis (DE). Images showed are the ones used for limiting the 3 ROIs in Figure 7 of the main text (n = 3) (scale bar = 50 µm).* (***B***) *From the pictures and values obtained, the skin crossing percentage was calculated and represented for each slide measured in each biological replicate (from different donors) (n = 3).* (***C***) *The mean value of each condition was used to calculate the crossing rate when compared to the control UreNMs in 0 mM urea in the viable epidermis, in the dermis and in total (combination of both).*

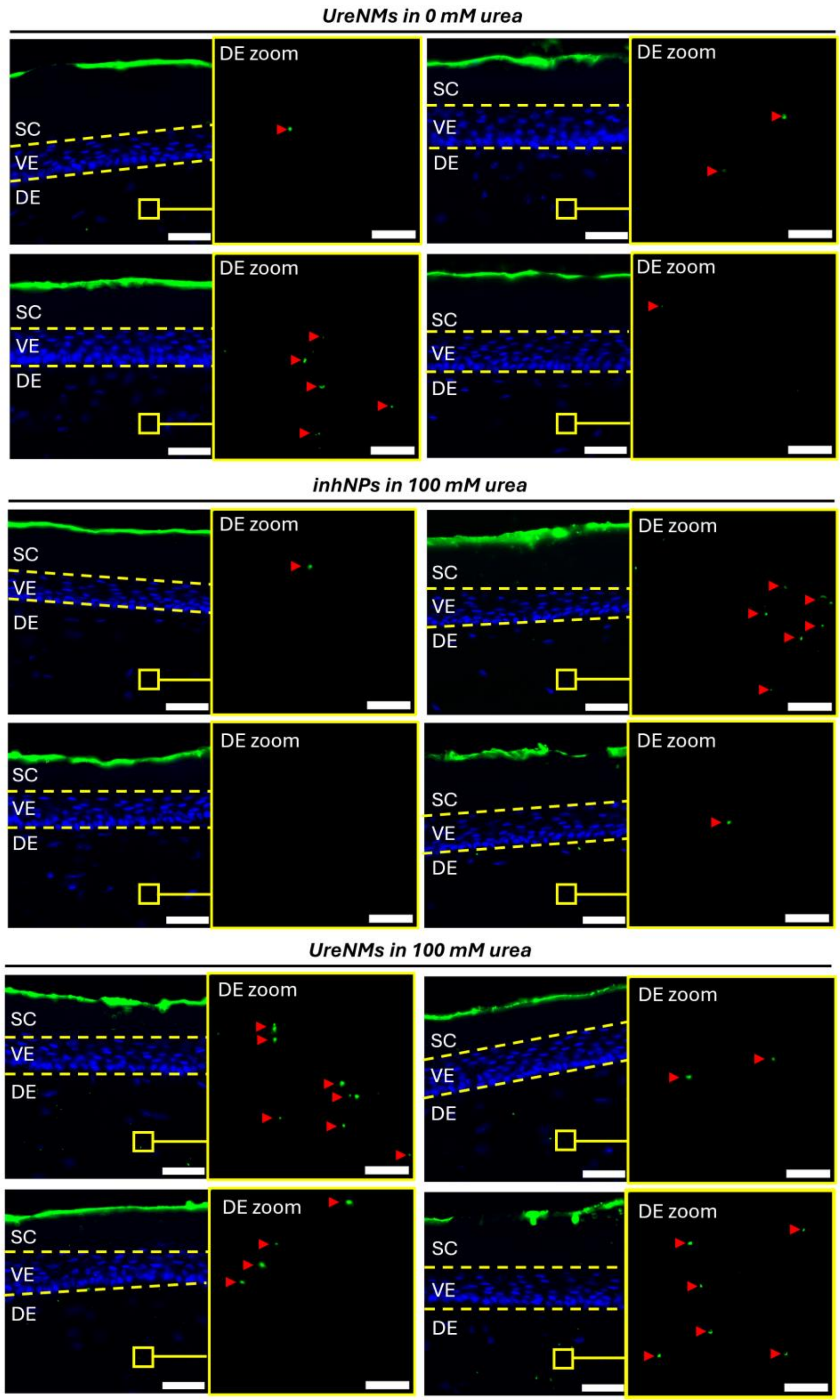

Figure S9. Human skin crossing studies. Same process as the one used in Supplementary Figure 8 was used to obtain more images of the skin models with inhNPs and UreNMs in 0 or 100 mM urea (scale bar = 50 µm) (SC = stratum corneum, VE = viable epidermis, DE = dermis). A zoomed image of the dermal layer was taken for each picture and the NPs detected indicated with a red arrow (DE zoom, scale bar = 15 µm).

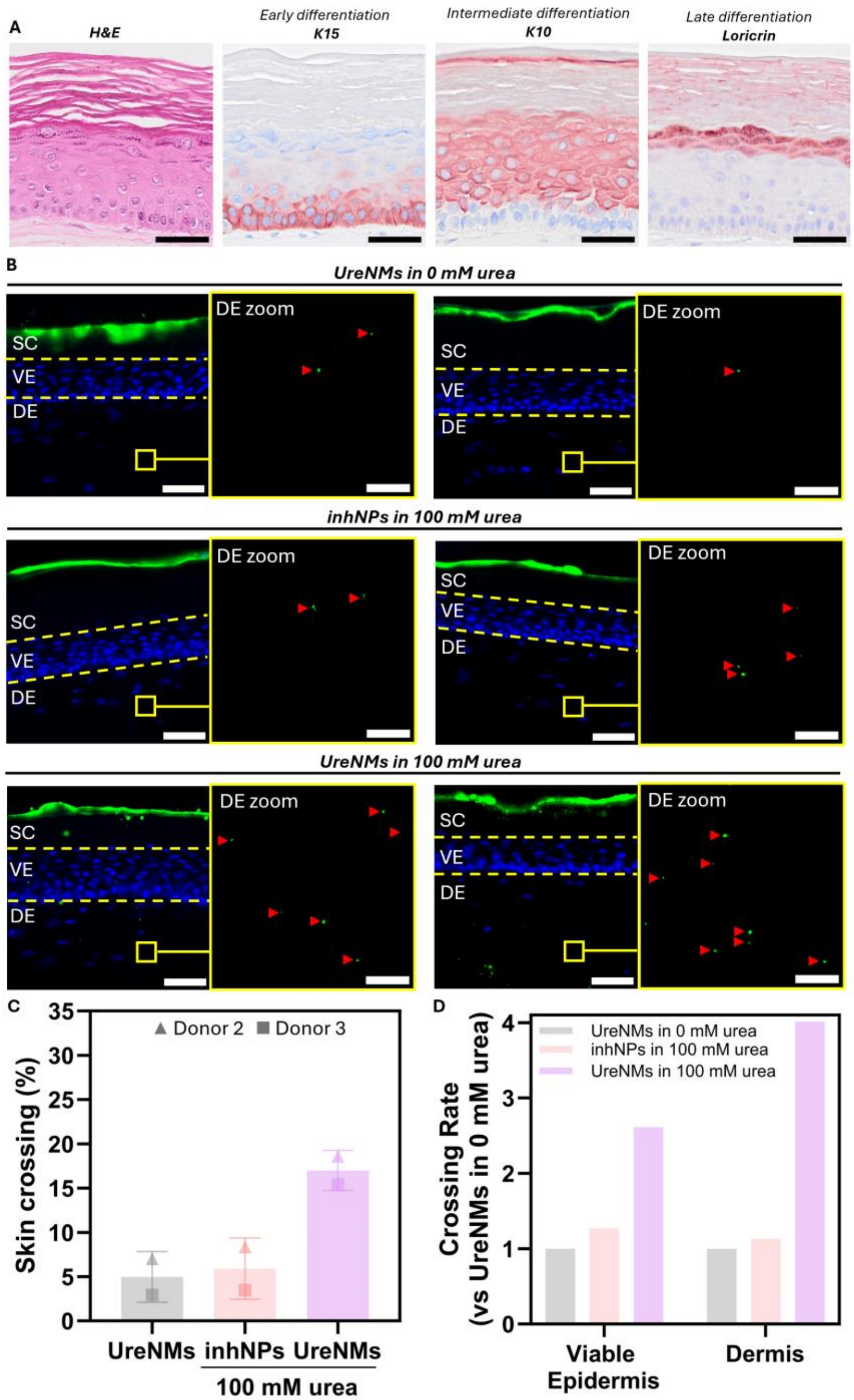

***Figure S10. Human skin crossing studies in lipid supplemented model**. (**A**) Human skin models supplemented with a lipid mixture were developed and characterized by H&E staining and immunohistochemistry against keratin 15 (K15, as basal layer marker), keratin 10 (K10, as differentiation suprabasal epidermis marker) and Loricrin (as terminal differentiation marker) (scale bar = 50 µm). (**B**) Cy5-labelled inhNPs and UreNMs (in green) were topically applied in 0 mM or 100 mM urea and incubated for 24 h. Samples were sectioned, stained with DAPI (cell nuclei in blue) and imaged with fluorescence microscopy (scale bar = 50 µm). The 3 ROIs (Stratum Corneum, viable epidermis and dermis) delimited with the yellow dotted lines were used to quantify the skin crossing percentage (viable epidermis + dermis) (**C**) and the mean values were used to calculate the crossing rate comparing with the control UreNMs in 0 mM urea (**D**) (n = 2). Results are shown as mean ± SD.*

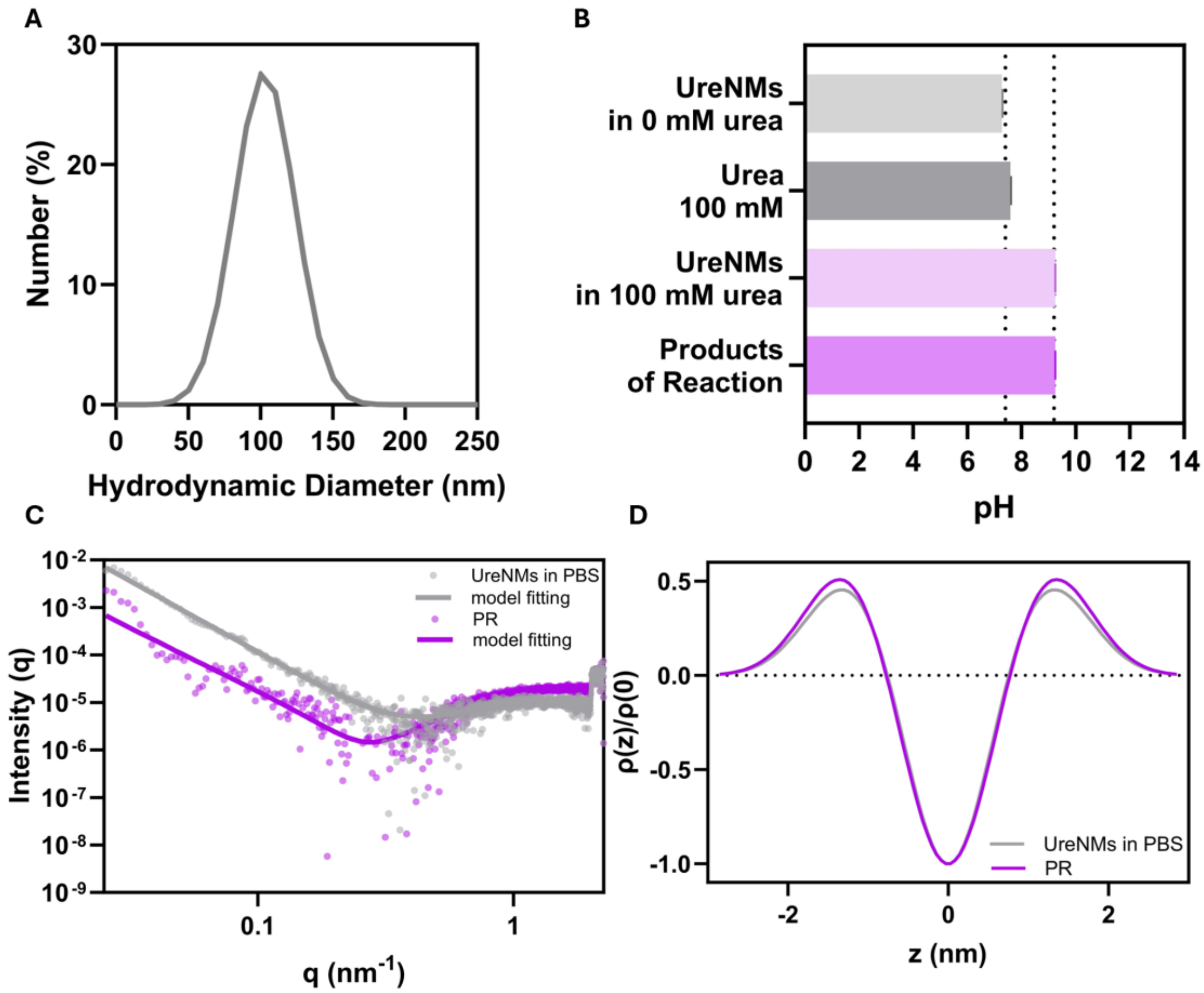

Figure S11. SAXS analysis of stratum corneum model. *(**A**) A membrane model mimicking the lipidic composition of stratum corneum was generated and characterize by DLS. (**B**) Urea 100 mM, UreNMs in absence and presence of urea and the products of the reaction (PR) were incubated 24h in PBS and the pH was measured (n = 3). Results are shown as mean ± SD. SAXS scattering plot of the SC models were obtained after incubation with the PR, fitted with a mathematical model assuming a bilayer structure (**C**) and transformed to electron density profile (**D**).*
